# A robust natural language text-to-SQL generation framework with dynamic strategies based on LLMs

**DOI:** 10.1038/s41598-026-39128-9

**Published:** 2026-02-09

**Authors:** Xiaodong Su, Yang Gu, Peng Wang, Wei Gu, Lincheng Qi, Jingwei He

**Affiliations:** 1 Lianyungang Power Supply Company, State Grid Jiangsu Electric Power Co., Ltd., Lianyungang, Jiangsu 222000 China; 2 Jiangsu Electric Power Information Technology Co., Ltd., Nanjing, Jiangsu 221000 China

**Keywords:** Computational biology and bioinformatics, Mathematics and computing

## Abstract

Natural language text-to-SQL generation (Text2SQL) aims to translate natural language questions into executable SQL queries. Although the emergence of large language models (LLMs) has led to significant advancements in this field, their performance degrades sharply with question complexity increases. A key limitation of current LLM-based methods lies in their uniform generation strategies, which fail to adapt dynamically to varying question complexity. To address this issue, we propose TriSQL, a novel three-stage framework designed to analyze question complexity and generate accurate and executable SQL. First, a Question-Guided Schema Selector is conceived to get the most relevant schema to the question using cross attention. Second, a Structure-Aware SQL Generator takes both the question and the selected schema as input, employing hierarchical decoding to generate a syntactically valid initial SQL. Finally, a Complexity-Aware SQL Refiner is designed with LLM to dynamically adjust strategies corresponding to the complexity of question and initial SQL, ensuring that the final generated SQL is both accurate and executable. Experimental results on the Spider benchmark and its variants show that TriSQL achieves state-of-the-art execution accuracy, surpasses existing LLM-based methods, and provides both high efficiency and strong robustness.

## Introduction

Domain-Specific Languages (DSLs) are specialized programming languages tailored to particular application domains, offering domain-aligned abstractions and well-defined grammars that improve expressiveness and reliability compared to general-purpose languages^1,2^. However, writing DSL programs still requires expertise in both the language syntax and the underlying domain. This creates a persistent usability gap for non-expert users who wish to interact with complex systems through high-level intent. Among DSLs, SQL is arguably the most widely used representative. It provides domain-specific constructs such as tables, joins, and aggregations, and exhibits a clear separation between structural scaffolding and domain content. These properties make SQL a natural and practical target for studying how to translate human intent into DSL programs.

Natural language text-to-SQL generation (Text2SQL) addresses this gap by translating user-issued natural language questions into executable SQL queries^3–6^. As a fundamental task in natural language processing (NLP), Text2SQL enables intuitive access to relational data and supports applications such as database question answering and business intelligence interfaces. Despite its apparent simplicity, an effective system must accurately capture user intent, ground mentions to the correct schema elements, and generate syntactically valid and semantically faithful SQL, especially under large schemas and complex query structures. Importantly, the role of SQL as a canonical DSL also makes Text2SQL an ideal testbed for techniques that may generalize to other query languages, including SQL dialects and non-relational alternatives such as MongoDB query language, Cypher for graph databases (Neo4j), or visual query interfaces for NoSQL data stores^7^.

Building on this formulation, prior Text2SQL research has progressed from sequence-to-sequence encoder-decoder models to structure-aware decoding and schema-centric representations. Early encoder-decoder approaches jointly encoded questions and schema tokens (tables and columns), often enhanced by schema linking features, and then generated SQL with attention and copy mechanisms^8,9^. Grammar-constrained decoding enforced syntactic validity through production rules, though sometimes at the cost of flexibility^10^. Relation-aware encoders, exemplified by RAT-SQL, modeled tables, columns, and foreign-key relations as structured graphs to strengthen question-schema grounding and improve compositional generalization^11^. These structure-aware ideas have further inspired broader semantic parsing efforts, including mapping natural language to graph query languages such as Cypher via heterogeneous graph representations^12^, and integrating relational structures with graph neural networks for multilingual and conversational parsing settings^13^. Other complementary directions include using intermediate sketches to simplify decoding, applying beam search with heuristic pruning, and introducing post-processing modules to fix type mismatches or syntactic errors^3,4^.

Although these methods achieved promising results, they showed limitations on large, complex databases. As the number of tables and columns increased, the accuracy of the generated SQL declined: string-level name matching was brittle to synonyms and abbreviations, and attention over irrelevant schemas introduced noise that led to incorrect joins and missing important tables or columns. Structural modeling remained fragile for complex SQL queries, as sequential decoders struggled to capture the hierarchical and compositional nature of SQL, which includes nested subqueries, long join paths, and intricate aggregations. Fixed grammar or template constraints reduced certain errors but either over-constrained the generation process or failed to cover atypical clause organizations. Strategies applied after SQL generation, such as type checking and parser-based correction, provide only marginal improvements. Since they are typically applied uniformly across all Text2SQL tasks, they rarely resolve deeper semantic errors and often introduce additional computational overhead.

Benefiting from the rapid progress of LLMs, a new wave of Text2SQL research has emerged, seeking to overcome these persistent limitations by leveraging the broad knowledge and strong reasoning capacity of LLMs. LLMs such as GPT-4, Codex, and LLaMA have accelerated progress in Text2SQL. Recent systems leverage them through in-context learning, supervised fine-tuning, and hybrid pipelines that couple LLMs with task-specific components, for example schema serialization and prompt templates that enumerate tables, columns, and textual descriptions, plus exemplars drawn from training logs or retrieved neighbors to steer generation toward the target domain^14–20^. Beyond vanilla prompting, several lines of enhancement have been explored. Dynamic interaction networks enhance the link between the question and the schema by using iterative message passing, which helps the model select the right tables and columns^21–23^. Action-based planning decomposes generation into a sequence of symbolic decisions such as selecting tables, composing joins, and instantiating predicates, which improves controllability and interpretability^24,25^. Retrieval-augmented decoding injects external evidence or schema-specific hints during generation and can delay retrieval to later steps to reduce noise^6,26–28^. Fast schema traversal prunes the search space with lightweight walks over foreign-key graphs or learned selectors so that the decoder operates on a compact, task-relevant schema view^29–31^. In practice, these ideas are often combined with grammar constraints, execution signals, and small post-hoc repair modules to curb syntax errors and improve executability^27,32^.

Despite these advances, current LLM-based Text2SQL methods still face critical challenges. First, as user questions become more complex, the accuracy and execution success of the generated SQL degrade significantly, showing the limited robustness of existing approaches^29,29–31,33^. Second, most approaches rely on training or fine-tuning with matching-based objectives that optimize string-level similarity to reference SQL queries. This emphasis on string matching can raise exact match scores but often produces SQL queries with structural defects and low execution accuracy, limiting their usefulness in practice^27,32,34^.

To overcome the above limitations, we present TriSQL, an LLM-based three-stage Text2SQL framework. In TriSQL, a Question-Guided Schema Selector selects the tables and columns from the database schema that are most relevant to the user question. By focusing only on these elements, it avoids interference from irrelevant schema parts and makes the following SQL generation more reliable. Next, a Structure-Aware SQL Generator applies hierarchical decoding from structure to content. It first produces the overall SQL skeleton and then gradually fills in the details. This approach gives high exact match scores while keeping the SQL structure valid, even for complex questions. Finally, a Complexity-Aware SQL Refiner uses an LLM to adjust generation strategies based on the complexity of the question and the initial SQL. The LLM then executes and iteratively refines the SQL on the database with feedback from the results. This ensures that the final SQL is both accurate and executable.

The main contributions of this work are as follows:

- We observe that current LLM-based Text-to-SQL methods mainly rely on string-level matching with gold SQL queries, which can raise exact match scores but does little to ensure executable accuracy. As a result, the generated SQL queries often contain structural errors, leading to complete failure on complex questions. This shows that existing methods lack robustness on complex questions, and motivates us to explore approaches that move beyond string-level matching.
- Motivated by this observation, we propose TriSQL, a three-stage LLM-based framework that addresses the limitations of existing methods. The framework integrates schema selection, structure-aware generation, and complexity-aware refinement into a unified pipeline, reducing the interference of irrelevant schema elements and ensuring both structural validity and execution reliability. Unlike prior approaches, TriSQL maintains high performance even on complex questions.
- Extensive experiments on large-scale Text2SQL benchmarks demonstrate that TriSQL consistently outperforms strong baselines, delivering substantial improvements in execution accuracy and showing greater performance on complex questions.

## Related work

### Context-aware Text2SQL generation methods

Sequence-to-Sequence (Seq2Seq) architectures have been widely adopted for the Text2SQL task due to their ability to directly map natural language questions to structured SQL queries^33,35,36^. Within this paradigm, attention mechanisms were introduced to highlight relevant parts of the input during decoding, improving alignment between natural language and schema elements^37,38^. These models usually encode the question together with a serialized version of the database schema and then decode the SQL token by token, often with copy mechanisms to help insert the correct tables and columns. Building on this foundation, subsequent research emphasized more effective use of contextual signals to improve schema linking and column selection. Some methods introduced specialized schema encoders or graph-based representations to capture table–column relationships and foreign-key dependencies, thereby providing richer context for the decoder^10,11^. Others explored intermediate sketch representations or type constraints to guide decoding toward more plausible SQL structures^4,39^. Editing-based methods treat SQL generation as iterative modifications of an existing SQL, enabling better handling of context in multi-turn or follow-up scenarios where previous interactions influence the current SQL^40–44^. RESDSQL^45^ decouples schema linking from SQL decoding, treating the identification of relevant tables and columns as a separate step before generation. This separation helps reduce interference during decoding, but the linking step largely relies on simplified relevance estimation between the question and schema items. As a result, it often ignores important semantic details in the question, which can introduce irrelevant tables or columns and miss critical ones needed for accurate SQL generation. Although these approaches often rely on coarse contextual signals and may include irrelevant or omit critical schema items, they nonetheless establish a solid foundation for Text2SQL by demonstrating the effectiveness of joint question–schema encoding. This shows the need for finer-grained, question-aware schema representations that link questions to the right tables and columns for SQL generation.

### Structure-aware Text2SQL generation methods

SQL queries inherently exhibit hierarchical and compositional structures, with major clauses such as SELECT, WHERE, and GROUP BY forming a skeleton that is further instantiated with tables, columns, and conditions. This property has motivated the development of structure-aware generation methods that move beyond flat token-by-token decoding. Early skeleton-based approaches exploited the observation that many SQL queries share similar structural templates, first constructing a high-level SQL skeleton and then instantiating it with specific tables, columns, and conditions^8,39,46–48^. These methods demonstrated that explicitly modeling the structure of SQL can improve both efficiency and accuracy.

Building on this intuition, later works introduced grammar-based constraints or Abstract Syntax Tree (AST) representations into the decoding process, ensuring syntactic validity and capturing nested structures such as subqueries and join hierarchies more faithfully^4,26,32^. Grammar-constrained decoding reduced the search space and guaranteed well-formed SQL, while AST-based methods provided a natural way to represent and generate SQL queries in a top-down manner. Complementing these structural approaches, recent efforts have explored incorporating refined grammatical information from natural language questions to better capture linguistic dependencies and semantic relationships^49^, demonstrating improvements particularly for complex and longer SQL queries where traditional graph neural networks struggle with grammatical complexity.

More recent efforts have attempted to combine these strategies with neural architectures or execution-guided signals to further mitigate syntax errors and improve logical consistency. Notably, hybrid approaches that merge different decoding paradigms have shown promise in addressing the individual limitations of sketch-based and generation-based methods^50^, offering improved syntactic accuracy while maintaining efficiency through simplified decoding processes and inter-SQL element modeling.

Despite these advances, structure-aware methods still face important limitations. Skeleton-based approaches often rely on predefined templates that cannot handle unconventional SQL queries. Grammar-constrained and AST-based methods can enforce syntactic correctness, but they are often too rigid, limiting flexibility and sometimes producing SQL queries that are correct in form but inconsistent with the user’s intent^3,3,51–53^. In addition, many existing methods handle structure and content separately during decoding, without considering their close relationship. To address these issues, our approach uses a structure-aware SQL generation module that models the hierarchical organization of SQL throughout decoding. This design preserves syntactic correctness while keeping enough flexibility to generate diverse and complex SQL queries, which improves robustness in practical applications.

### LLM-based Text2SQL methods

The rapid development of LLMs has brought new opportunities for Text2SQL, as these models acquire broad knowledge and reasoning ability through large-scale pretraining^10,54–56^. They can follow natural language instructions and reason across different contexts, which makes them appealing for directly converting user questions into SQL. However, prompting LLMs to generate SQL alone often gives limited accuracy, particularly on complex or domain-specific databases. The main difficulties are linking questions to the correct schema elements, keeping the generated SQL structurally correct, and handling complex SQL logic such as multiple joins, nested subqueries, and aggregations.

Several recent studies have attempted to mitigate these issues while still treating LLMs as a standalone generator. For example, DIN-SQL^21^ improves generation by adding a schema linking step to the prompt, guiding the LLM with difficulty-aware instructions. Kang and Wang^57,58^ further refine prompt design by embedding schema knowledge into reference-based templates, demonstrating that careful prompt construction can enhance LLM performance even without full end-to-end supervision. Beyond general-purpose applications, domain-specific adaptations have emerged to address specialized requirements: innovative approaches have been developed for financial industry applications by incorporating Python-based processing with large language models^19^, while spatial database SQL has been enhanced through GPT-based methods that integrate geographic and spatial knowledge directly into prompts, requiring only minimal training examples^20^.Similarly, efforts to adapt and evaluate LLMs for low-resource languages have led to the creation of new benchmarks, such as TURSpider for Turkish, to measure and improve model performance outside of English-centric domains.^59^

While these methods use the reasoning ability of LLMs, they still depend on handcrafted prompt engineering and mostly treat the model as a standalone generator^10,11,60,61^. This dependence makes them sensitive to prompt design and less reliable when generating SQLs of different complexity. In contrast, our framework moves away from the standalone setting by using the LLM in a more specific role, as a Complexity-Aware SQL Refiner within a three-stage pipeline. With support from schema selection and structure-aware generation, TriSQL enables the LLM to concentrate on refining SQLs generated from complex questions.

Beyond relational SQL, recent work has started to investigate natural language interfaces for NoSQL databases, where weakly defined or evolving schemas and semi-structured data introduce additional challenges. MTable^7^ suggests that visual query interfaces can support exploration in such settings by exposing document structure and key-value relationships in an interpretable form. In contrast, direct natural language translation to NoSQL query languages remains relatively underexplored, in part due to the absence of large-scale, standardized benchmarks. Nevertheless, core principles from Text-to-SQL, such as schema-aware grounding, structure-first generation, and execution-guided refinement, provide a useful starting point.

## Proposed methodology

### Analysis of Text2SQL model robustness

Robustness to increasing query complexity is a key requirement for deploying Text2SQL systems in real-world analytics. In practice, user questions vary substantially in both structural complexity (e.g., nested queries, multi-table joins, compositional operators) and semantic complexity (e.g., ambiguous constraints, implicit conditions, long-range dependencies). As a result, a model that performs well on easier questions can still be unreliable in realistic settings if its execution accuracy degrades sharply as questions become more complex. This motivates an explicit robustness analysis: not only reporting a single aggregate score, but also evaluating how well a model sustains executable semantic correctness under progressively harder question patterns. Such an analysis directly supports the motivation of this paper.

We analyze model robustness under increasing structural and semantic question complexity. We define robustness as a model’s ability to maintain high execution accuracy with minimal performance degradation as complexity increases. Let $\mathscr {C} = \{c_1, c_2, \ldots , c_K\}$ denote a set of *K* complexity levels ordered by increasing difficulty, where in our evaluation $\mathscr {C} = \{\textsf{low}, \textsf{medium}, \textsf{high}, \mathsf {extra\text {-}high}\}$ with $K=4$. For a given model $\mathscr {M}$ and complexity level $c_k \in \mathscr {C}$, let $\textrm{EX}(\mathscr {M}, c_k)$ denote the execution accuracy at that level. We define the robustness score $\mathscr {R}(\mathscr {M})$ as:

1 $$\begin{aligned} \mathscr {R}(\mathscr {M}) = \frac{1}{K}\sum _{k=1}^{K} \textrm{EX}(\mathscr {M}, c_k)\;-\;\frac{1}{K-1}\sum _{k=1}^{K-1}\big [\textrm{EX}(\mathscr {M}, c_k)-\textrm{EX}(\mathscr {M}, c_{k+1})\big ]. \end{aligned}$$

The first term measures average execution accuracy across all complexity levels. The second term measures the average degradation between consecutive complexity levels. A more robust model achieves higher overall execution accuracy while exhibiting smaller drops as complexity increases.

**Fig. 1:**
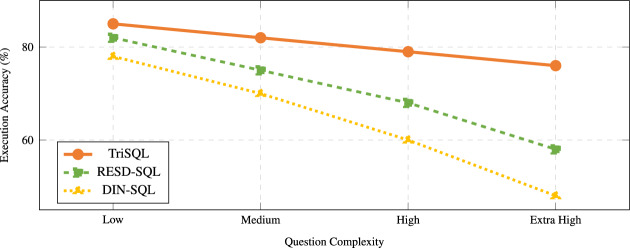
Performance trends under increasing question complexity. TriSQL maintains higher accuracy and degrades more slowly than competing methods.

Figure 1 shows execution accuracy trends across complexity levels for TriSQL and two strong baselines. Although all methods experience performance decline as complexity increases, TriSQL exhibits superior robustness with both higher average accuracy and slower degradation. Specifically, TriSQL achieves $\mathscr {R}(\text {TriSQL}) = 0.805 - 0.060 = 0.745$ with average EX of 80.5% and average degradation of 6.0 percentage points between consecutive levels. In contrast, RESDSQL obtains $\mathscr {R}(\text {RESDSQL}) = 0.708 - 0.107 = 0.601$ with average EX of 70.8% and degradation of 10.7 percentage points, while DIN-SQL achieves $\mathscr {R}(\text {DIN-SQL}) = 0.640 - 0.133 = 0.507$ with average EX of 64.0% and degradation of 13.3 percentage points. These robustness scores quantitatively confirm that TriSQL maintains more stable performance under increasing complexity.

At the extra-high complexity level, TriSQL achieves 76% execution accuracy compared to 58% for RESDSQL and 48% for DIN-SQL, demonstrating substantially better robustness on the hardest questions. These results also reveal a key limitation of existing methods. Many emphasize string-level matching to improve exact match scores, which can overestimate correctness. Under complex structural and semantic patterns, such methods often produce SQL that appears similar to references but fails to execute with the intended meaning, leading to sharp losses in execution accuracy and reduced robustness.

While exact match measures literal similarity between predicted and gold SQL, execution accuracy evaluates whether the generated query retrieves the correct results and matches the question intent, even when multiple syntactic forms are valid. Using execution accuracy across complexity bins therefore provides a direct and practical measure of robustness, since it reflects semantic correctness under harder compositional patterns. These observations motivate approaches that prioritize stable executable semantics via accurate schema linking, structure-aware generation, and adaptive refinement, to keep performance reliable as SQL complexity grows. Based on this motivation, we develop TriSQL, an LLM-based three-stage Text2SQL framework designed to improve robustness under complex questions.

### Overall design of TriSQL

We present TriSQL, an LLM-based three-stage Text-to-SQL framework that generates executable SQL queries through three specialized stages. Because SQL queries differ in structural complexity and databases vary widely in schema design, using a single uniform strategy for all inputs often leads to poor performance, especially on complex cases. To address this, TriSQL first selects the schema elements most relevant to the question, then generates SQL queries with clear structure and valid syntax and semantics, and finally refines them through complexity-aware reasoning. This step-by-step design improves both accuracy and efficiency by adapting the generation process to the complexity of each question and the diversity of database schemas.

**Fig. 2:**
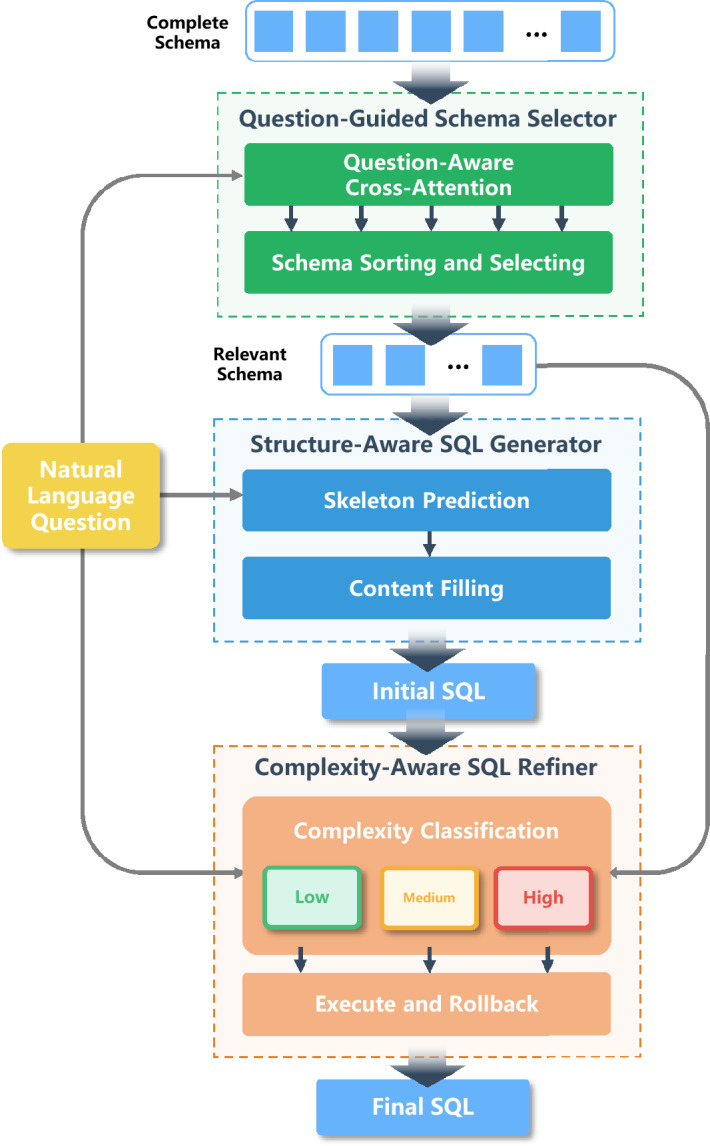
Overall architecture of TriSQL. The framework has three sequential stages: a Question-Guided Schema Selector that selects schema elements most relevant to the input question, a Structure-Aware SQL Generator that uses the hierarchical structure of SQL to produce syntactically and structurally correct SQL queries, and a Complexity-Aware SQL Refiner that makes targeted modifications to SQL queries based on the complexity of the natural language question and the initial SQL output.

The framework contains three components: a Question-Guided Schema Selector, a Structure-Aware SQL Generator, and a Complexity-Aware SQL Refiner. Each component plays a different role in the pipeline. The Question-Guided Schema Selector selects schema elements that are most relevant to the input question, reducing the effect of unrelated parts and keeping the downstream generation focused on what is essential. The Structure-Aware SQL Generator captures the hierarchical structure of SQL and produces SQL queries that are structurally correct while remaining flexible for different SQL forms. The Complexity-Aware SQL Refiner improves these SQL queries by adapting its refinement according to the complexity of the question and the initial SQL, which is especially useful in complex cases. Figure 2 gives an overview of the framework, showing how the three components work together step by step to produce executable SQL queries.

### Question-guided schema selector

Large database schemas often include many tables and columns that are unrelated to the user question. Using these irrelevant elements in SQL generation introduces noise and lowers accuracy. Existing schema selection methods usually depend on coarse relevance scores or static schema encodings, which may keep irrelevant elements or miss subtle but important links between the question and the schema. These problems become more serious in complex SQL queries, where even one unnecessary table or column can mislead the generation. To solve this, we introduce a Question-Guided Schema Selector that makes question-aware, fine-grained selections of schema elements, resulting in a smaller and more relevant schema subset for downstream SQL generation.

Given a natural language question *q*, the complete database schema is denoted as $S = \{T_1, T_2, \dots , T_n\}$, where each table $T_i$ is associated with a set of columns $C_i = \{C_i^1, C_i^2, \dots , C_i^{m_i}\}$. To estimate the relevance of schema elements with respect to the question, we apply cross-attention in two stages. The table-level relevance scores are:

2 $$\begin{aligned} w^{(t)}_i = \textrm{softmax}\!\left( \textrm{Attn}(q, T_i)\right) , \quad i=1,\dots ,n, \end{aligned}$$

where $\textrm{Attn}(q, T_i)$ denotes the cross-attention score between *q* and table $T_i$. Similarly, column-level relevance scores within each table are computed as follows:

3 $$\begin{aligned} w^{(c)}_{ij} = \textrm{softmax}\!\left( \textrm{Attn}(q, C_i^j)\right) , \quad j=1,\dots ,m_i, \end{aligned}$$

where $\textrm{Attn}(q, C_i^j)$ is the cross-attention score between *q* and column $C_i^j$. Finally, the relevance of the table is refined by aggregating the relevance of its columns as follows:

4 $$\begin{aligned} \tilde{w}^{(t)}_i = w^{(t)}_i + \lambda \sum _{j=1}^{m_i} w^{(c)}_{ij}, \end{aligned}$$

where $\lambda$ is a trade-off parameter that controls the contribution of column-level information.

We then determine the filtered schema subset by thresholding the final table relevance weights:

5 $$\begin{aligned} S_{\text {ctx}} = \{\, T_i \mid \tilde{w}^{(t)}_i \ge \tau \,\}, \end{aligned}$$

where $\tau$ is a predefined threshold. This operation yields the minimal yet sufficient set of tables and their associated columns for accurate SQL generation, thereby reducing search space and improving the effectiveness of subsequent stages in the framework.

**Algorithm 1:**
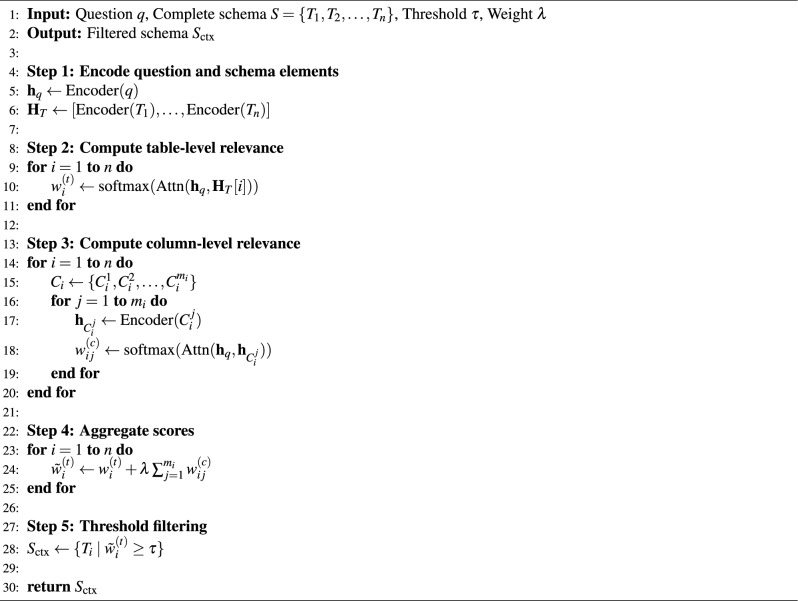
Question-Guided Schema Selector

To make the above scoring and filtering procedure concrete and easy to reproduce, we summarize the complete schema selection workflow in Algorithm 1. It implements question-guided schema selection through five steps. Step 1 encodes the question *q* and all table names into dense representations using a pretrained encoder, ensuring that semantically similar terms are mapped to nearby embeddings. Step 2 computes table-level relevance scores $w^{(t)}_i$ via cross-attention, identifying which tables are mentioned or implied by the question. Step 3 computes column-level relevance scores $w^{(c)}_{ij}$ within each table, capturing fine-grained relevance that may be missed by table names alone. Step 4 aggregates table-level and column-level scores, where the hyperparameter $\lambda$ controls the contribution of column information. Our sensitivity analysis (Fig. 10) shows that $\lambda = 0.8$ provides optimal balance. Step 5 applies threshold filtering to produce the filtered schema subset $S_{\text {ctx}}$, with $\tau = 0.6$ balancing recall and precision based on grid search (Fig. 11).

### Structure-aware SQL generator

Accurately generating complex SQL queries requires not only aligning with the meaning of the input question but also explicitly modeling the hierarchical structure of SQL. To achieve this, the Structure-Aware SQL Generator uses a two-phase decoding process. In the first phase, it predicts the overall SQL structure by specifying the main clauses (e.g., SELECT, WHERE, GROUP BY) and their order, and inserts typed placeholders to mark positions that must be instantiated with schema-specific content. During training, these placeholder positions are supervised using human-annotated masks derived from the ground-truth SQL, allowing the model to learn both their locations and quantity. At inference time, the model therefore infers the placeholder layout directly from the input question and the filtered schema context, without any manual configuration. Simpler questions typically induce fewer placeholders, whereas complex queries with multiple joins, nested conditions, or aggregations require more. In the second phase, the model fills these placeholders with tables, columns, join conditions, and constants from the filtered schema subset $S_{\text {ctx}}$ provided by the Question-Guided Schema Selector.

Formally, let $y_{\text {struct}}$ denote the sequence of structural tokens generated in the first phase. Its probability is modeled as:

6 $$\begin{aligned} P_{\text {struct}}(y_{\text {struct}} \mid q, S_{\text {ctx}}) = \prod _{t=1}^{|y_{\text {struct}}|} P(y^{(t)}{\text {struct}} \mid y^{(<t)}{\text {struct}}, q, S_{\text {ctx}}), \end{aligned}$$

where *q* is the input question and $S_{\text {ctx}}$ is the filtered schema subset.

Once the structure is obtained, the second phase fills each placeholder $p_k$ in $y_{\text {struct}}$ with content tokens from $S_{\text {ctx}}$, producing the final SQL $y_{\text {sql}}$:

7 $$\begin{aligned} P_{\text {sql}}(y_{\text {sql}} \mid y_{\text {struct}}, q, S_{\text {ctx}}) = \prod _{k=1}^{K} P(c_k \mid p_k, y_{\text {struct}}, q, S_{\text {ctx}}), \end{aligned}$$

where ${c_k}_{k=1}^K$ are the schema elements or constants assigned to the *K* placeholders.

To better illustrate this progressive decomposition, Table 1 presents an example showing how the framework first establishes a coherent structure and then incrementally fills in schema-specific details. By explicitly separating structure prediction from content filling, the Structure-Aware SQL Generator maintains global structural integrity while producing clause-level content that is semantically faithful to the input question. The output of this stage is a SQL query with a clear and valid structure, which is then passed to the Complexity-Aware SQL Refiner for further modification.

**Table 1:** SQL generation in the Structure-Aware SQL Generator. Given the question *q*, the Question-Guided Schema Selector provides the filtered schema $S_{\text {ctx}}$. The generator predicts a structural plan $y_{\text {struct}}$ that specifies the overall clause organization, and then instantiates it with elements from $S_{\text {ctx}}$ to produce the final SQL $y_{\text {sql}}$.

Question (*q*)	Find all employees in the engineering department who have been with the company for more than 5 years, along with their current salaries.
**Filtered Schema** ($S_{\text {ctx}}$)	Tables: employees(e_id, name, hire_date, dept_id); departments(dept_id, dept_name); salaries(emp_id, salary).
**Structural Plan** ($y_{\text {struct}}$)	SELECT $\texttt {<}$fields$\texttt {>}$ FROM employees JOIN departments ON $\texttt {<}$dept_join$\texttt {>}$ JOIN salaries ON $\texttt {<}$emp_join$\texttt {>}$ WHERE $\texttt {<}$dept_constraint$\texttt {>}$ AND $\texttt {<}$tenure_constraint$\texttt {>}$
**Instantiated SQL** ($y_{\text {sql}}$)	SELECT e.name, e.hire_date, s.salary FROM employees e JOIN departments d ON e.dept_id = d.dept_id JOIN salaries s ON e.emp_id = s.emp_id WHERE d.dept_name = ’engineering’ AND DATEDIFF(CURDATE(), e.hire_date) $\texttt {>}$ 1825

**Algorithm 2:**
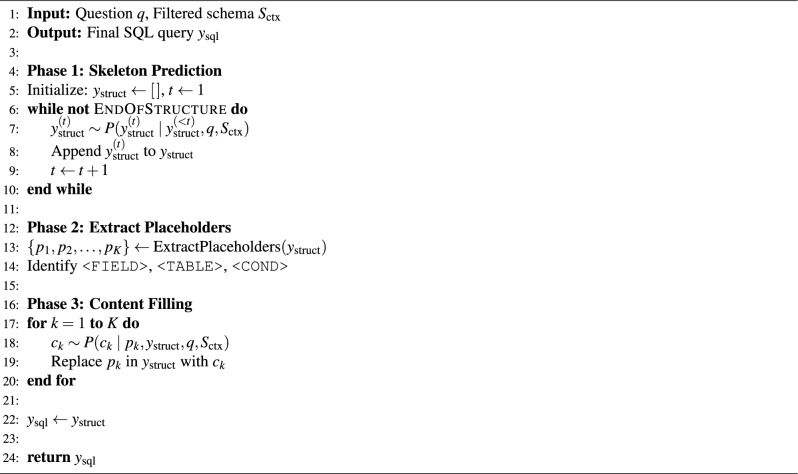
Structure-aware SQL generator.

To make this process explicit and reproducible, we summarize the full generation workflow in Algorithm 2. It implements two-phase SQL generation. Phase 1 generates a structural skeleton $y_{\text {struct}}$ through autoregressive decoding, producing SQL keywords and typed placeholders. Phase 2 extracts the typed placeholders from $y_{\text {struct}}$ to form an ordered placeholder list. Phase 3 fills each placeholder $p_k$ with concrete schema elements or values from the filtered schema $S_{\text {ctx}}$.

This design ensures structural validity via skeleton-first planning, reducing clause-ordering and syntax errors. It also ensures semantic grounding through question-aware and schema-aware instantiation, and compositional generalization to unseen schema combinations.

### Complexity-aware SQL refiner

The final stage refines the SQL generated by the previous module using both the natural language question *q* and the relevant schema $S_{\text {ctx}}$. Instead of applying the same process to all SQLs, the refiner adapts its strategy based on an estimated complexity level derived from $(q, y_{\text {sql}}, S_{\text {ctx}})$. In this way, it focuses on refinement when the SQL is complex and needs structural or semantic fixes, while avoiding unnecessary changes for simpler cases.

Formally, a classifier $f_{\text {cls}}$ maps the triplet $(q, y_{\text {sql}}, S_{\text {ctx}})$ to a complexity level:

8 $$\begin{aligned} z = f_{\text {cls}}(q, y_{\text {sql}}, S_{\text {ctx}}), \quad z \in \{\textsf{low}, \textsf{medium}, \textsf{high}\}, \end{aligned}$$

where the profile *z* reflects structural, semantic and relational complexity indicators such as the number of joined tables, nesting depth, and constraint types. In practice, $f_{\text {cls}}$ is implemented using a BERT encoder followed by a fully connected network, which is able to capture rich semantic dependencies between the question *q*, the candidate SQL $y_{\text {sql}}$, and the relevant schema $S_{\text {ctx}}$, providing an effective assessment of the overall complexity.

Once the complexity level *z* is determined, the Complexity-Aware SQL Refiner allocates the refinement effort proportionally. Question and initial SQL at the low level ($z=\textsf{low}$) are only given minor corrections, such as fixing simple syntax errors or adjusting keywords. Question and initial SQL at the medium level ($z=\textsf{medium}$) receive structural adjustments to improve clause organization and ensure consistency with $S_{\text {ctx}}$. And question and initial SQL at the high level ($z=\textsf{high}$) are subjected to fine-grained reasoning and decomposition-based modifications guided by the LLM. This adaptive design ensures that the refinement effort is in accordance with the assessed complexity level before any execution validation is performed. We denote the initial SQL generated by the Structure-Aware SQL Generator as $y_{\text {sql}}^{\text {init}}$, the refined SQL as $y_{\text {sql}}^{\text {ref}}$, and the final output as $y_{\text {sql}}^{\text {out}}$. Execution validation is then applied to each candidate:

9 $$\begin{aligned} V(y_{\text {sql}}^{*}) = \textsf{Exec}(y_{\text {sql}}^{*}, \mathscr {D}, \tau ), \end{aligned}$$

where $y_{\text {sql}}^{*} \in \{y_{\text {sql}}^{\text {init}}, y_{\text {sql}}^{\text {ref}}\}$, $\mathscr {D}$ is the database, and $\tau$ is the maximum time limit. The function $V(\cdot )$ records execution success, runtime, and basic output statistics. Based on these results, a decision function determines whether refinement should be accepted:

10 $$\begin{aligned} d = f_{\text {assess}}\big (y_{\text {sql}}^{\text {init}}, V(y_{\text {sql}}^{\text {init}}), y_{\text {sql}}^{\text {ref}}, V(y_{\text {sql}}^{\text {ref}}), q, z\big ), \end{aligned}$$

where $f_{\text {assess}}$ considers execution validity, how well the SQL matches the question *q*, the predicted complexity level *z*, and the relative quality of the initial and refined SQLs. If refinement is needed, the LLM updates $y_{\text {sql}}^{\text {init}}$ into $y_{\text {sql}}^{\text {ref}}$ using prompts adapted to the assessed complexity level. As shown in Table 2, this process can turn a non-executable SQL into one that runs correctly.

**Table 2:** Example of complexity-aware refinement. Given the question *q* and the relevant schema $S_{\text {ctx}}$, the Complexity-Aware SQL Refiner adapts to the medium complexity level ($z=\textsf{medium}$) and applies structural adjustments. As a result, the initial non-executable SQL $y_{\text {sql}}^{\text {init}}$ is turned into the refined SQL $y_{\text {sql}}^{\text {ref}}$, which can be executed correctly and yields better results.

**Question** (*q*)	Find all employees in the Engineering department who have worked on more than 2 projects in the last year, along with their total number of projects and average project budget.
**Relevant Schema** ($S_{\text {ctx}}$)	**Tables:** employees (emp_id, name, dept_id, hire_date, salary), departments (dept_id, dept_name, manager_id), projects (proj_id, proj_name, start_date, end_date, budget), assignments (emp_id, proj_id, assigned_date, role)
	**Foreign Keys:** employees.dept_id $\rightarrow$ departments.dept_id; assignments.emp_id $\rightarrow$ employees.emp_id
**Initial SQL** ($y_{\text {sql}}^{\text {init}}$)	SELECT e.name, COUNT(a.proj_id) AS project_count
	FROM employees e
	JOIN departments d ON e.dept_id = d.dept_id
	JOIN assignments a ON e.emp_id = a.emp_id
	WHERE d.dept_name = ’engineering’
	AND a.assigned_date $\texttt {>}$= ’2023-01-01’
	GROUP BY e.name
	HAVING COUNT(a.proj_id) $\texttt {>}$ 2
	***Status:*** $V(y_{\text {sql}}^{\text {init}}).\text {exec} = 0$ (Non-executable)
	***Issues:*** Missing average budget calculation; case sensitivity error
**Complexity Level** (*z*)	$z = \textsf{medium}$
	**Structural:** Multi-table joins (3 tables), GROUP BY with HAVING
	**Semantic:** Aggregation requirements (COUNT, AVG), temporal constraints
	**Relational:** Cross-table dependencies, foreign key navigation
	**Refinement Strategy:** Structural optimization with guided join relationships
**LLM Refinement Guidance**	$\bullet$ Fix case sensitivity for department name matching
	$\bullet$ Add missing average budget calculation via projects table
	$\bullet$ Ensure proper temporal constraint handling
	$\bullet$ Optimize join order and add result ordering
**Refined SQL** ($y_{\text {sql}}^{\text {ref}}$)	SELECT e.name, COUNT(DISTINCT a.proj_id) AS project_count,
	AVG(p.budget) AS avg_budget
	FROM employees e
	JOIN departments d ON e.dept_id = d.dept_id
	JOIN assignments a ON e.emp_id = a.emp_id
	JOIN projects p ON a.proj_id = p.proj_id
	WHERE LOWER(d.dept_name) = ’engineering’
	AND a.assigned_date $\texttt {>}$= DATE(’now’, ’-1 year’)
	GROUP BY e.emp_id, e.name
	HAVING COUNT(DISTINCT a.proj_id) $\texttt {>}$ 2
	ORDER BY project_count DESC
	**Status:** $V(y_{\text {sql}}^{\text {ref}}).\text {exec} = 1$ (Executable)
	**Quality Score:** $Q(y_{\text {sql}}^{\text {ref}}) = 94/100$

In practical deployment, we observed that refinement occasionally introduces new errors or fails to improve the SQL quality. To address this issue and enhance robustness, we design an adaptive fallback strategy:

11 $$\begin{aligned} y_{\text {sql}}^{\text {out}} = {\left\{ \begin{array}{ll} y_{\text {sql}}^{\text {ref}}, & V(y_{\text {sql}}^{\text {ref}}).\text {exec} \wedge \lnot V(y_{\text {sql}}^{\text {init}}).\text {exec},\\ y_{\text {sql}}^{\text {init}}, & V(y_{\text {sql}}^{\text {init}}).\text {exec} \wedge \lnot V(y_{\text {sql}}^{\text {ref}}).\text {exec},\\ \arg \max \limits _{y \in \{y_{\text {sql}}^{\text {init}},\, y_{\text {sql}}^{\text {ref}}\}} Q(y), & \text {if both executable},\\ \text {Refiner}\big (q, y_{\text {sql}}^{\text {init}}, S_{\text {ctx}}, z{+}1\big ), & \lnot V(y_{\text {sql}}^{\text {init}}).\text {exec} \wedge \lnot V(y_{\text {sql}}^{\text {ref}}).\text {exec} \wedge z < \textsf{high},\\ \text {UserError}, & \lnot V(y_{\text {sql}}^{\text {init}}).\text {exec} \wedge \lnot V(y_{\text {sql}}^{\text {ref}}).\text {exec} \wedge z = \textsf{high}. \end{array}\right. } \end{aligned}$$

Here *Q*(*y*) denotes the quality evaluation function that combines multiple signals, including semantic alignment with the question *q*, consistency of execution results, and structural plausibility with respect to $S_{\text {ctx}}$. If both candidates are executable, the one with higher *Q*(*y*) is selected. If neither candidate is executable, the system escalates by increasing the complexity level *z* and re-invoking the refiner; if refinement still fails at $z=\textsf{high}$, the system issues an explicit error message to the user instead of returning an invalid SQL.

This procedure applies refinement mainly to the SQLs that need it most, while validation and fallback keep the process reliable. As a result, execution accuracy improves greatly on high-complexity SQLs, with only small overhead on simple ones. Ablation studies in our experiments further show that complexity-aware refinement is effective under these controls.

**Table 3:** Example of fallback with complexity escalation. Given the question *q* and the relevant schema $S_{\text {ctx}}$, both the initial SQL $y_{\text {sql}}^{\text {init}}$ and the first refined SQL $y_{\text {sql}}^{\text {ref}}$ fail to execute. The framework then escalates to $z=\textsf{high}$ and produces a refined SQL that runs successfully as the final output $y_{\text {sql}}^{\text {out}}$. This shows how escalation helps avoid invalid SQL and ensures a correct executable result.

Question (*q*)	List the names of employees who managed projects with a budget exceeding $1M but had no direct reports in their department.
**Relevant Schema** ($$S_{\text {ctx}}$$)	**Tables:** employees (emp_id, name, dept_id, manager_id), departments (dept_id, dept_name), projects (proj_id, proj_name, budget, manager_id), assignments (emp_id, proj_id)
	**Foreign Keys:** employees.dept_id $$\rightarrow$$ departments.dept_id; projects.manager_id $$\rightarrow$$ employees.emp_id; assignments.emp_id $$\rightarrow$$ employees.emp_id
**Initial SQL** ($$y_{\text {sql}}^{\text {init}}$$)	SELECT e.name
	FROM employees e JOIN projects p ON e.emp_id = p.manager_id
	WHERE p.budget $$\texttt {>}$$ 1000000 AND e.manager_id IS NULL
	***Status:*** $$V(y_{\text {sql}}^{\text {init}}).\text {exec} = 0$$ (Non-executable)
	***Error:*** Invalid logic for detecting “no direct reports”
**Refined SQL **($$y_{\text {sql}}^{\text {ref}}$$)	SELECT e.name
	FROM employees e JOIN projects p ON e.emp_id = p.manager_id
	LEFT JOIN employees r ON e.emp_id = r.manager_id
	WHERE p.budget $$\texttt {>}$$ 1000000 AND r.emp_id IS NULL
	***Status:*** $$V(y_{\text {sql}}^{\text {ref}}).\text {exec} = 0$$ (Non-executable)
	***Error:*** Ambiguous join conditions
**Fallback Decision**	**Decision Logic:** Both $$y_{\text {sql}}^{\text {init}}$$ and $$y_{\text {sql}}^{\text {ref}}$$ fail execution.
	Applied Rule: $$\lnot V(y_{\text {sql}}^{\text {init}}).\text {exec} \wedge \lnot V(y_{\text {sql}}^{\text {ref}}).\text {exec}$$
	Action: escalate complexity level ($$z \rightarrow z{+}1$$) and re-invoke refinement.
**Escalated Refinement** ($$z=\textsf{high}$$)	SELECT e.name
	FROM employees e JOIN projects p ON e.emp_id = p.manager_id
	WHERE p.budget $$\texttt {>}$$ 1000000 AND NOT EXISTS
	(SELECT 1 FROM employees r WHERE r.manager_id = e.emp_id)
	**Status:** $$V(y_{\text {sql}}^{\text {ref}}).\text {exec} = 1$$ (Executable)
	**Quality Score:** $$Q(y_{\text {sql}}^{\text {ref}}) = 92/100$$
**Final Output** ($$y_{\text {sql}}^{\text {out}}$$)	$$y_{\text {sql}}^{\text {ref}}$$ from escalated refinement ($$z=\textsf{high}$$).
	**Status:** Executable
	**Rationale:** Complexity escalation successfully produced a valid SQL.

**Algorithm 3:**
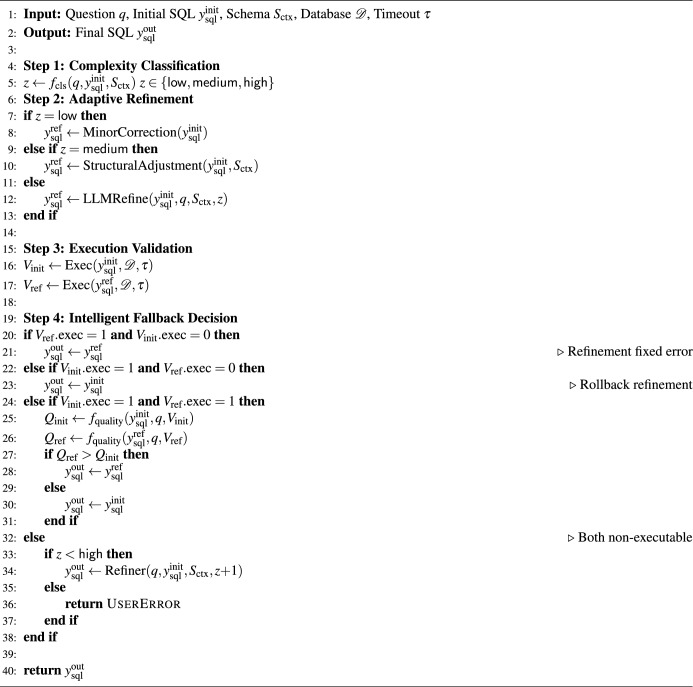
Complexity-aware SQL refiner.

To operationalize the above refinement strategy and make the decision flow explicit, we summarize the complete adaptive refinement pipeline in Algorithm 3. It implements adaptive refinement through four steps. Step 1 classifies the complexity level $z \in \{\textsf{low}, \textsf{medium}, \textsf{high}\}$ using a BERT-based classifier $f_{\text {cls}}$) trained on structural features, semantic features, and relational features. Step 2 applies complexity-aware refinement. Low-complexity cases invoke MinorCorrection for lightweight syntax fixes. Medium-complexity cases apply StructuralAdjustment to reorganize clauses, convert implicit joins into explicit JOIN syntax, and verify GROUP BY consistency using schema constraints from $S_{\text {ctx}}$. High-complexity cases prompting a large language model with the question, the initial SQL, the schema, and detected errors to perform deeper semantic refinement. Step 3 validates both $y_{\text {sql}}^{\text {init}}$ and the refined query $y_{\text {sql}}^{\text {ref}}$ by executing them on the database $\mathscr {D}$ (Eq. (8)), recording execution status, runtime, and result metadata. Step 4 implements intelligent fallback. If only the refined SQL executes successfully, refinement is accepted. If only the initial SQL executes, the system rolls back to avoid degradation. When both execute, the version with the higher quality score $Q(\cdot )$, computed from semantic alignment, structural plausibility, and execution efficiency, is selected. When neither executes, the algorithm escalates the complexity level ($z \leftarrow z+1$) and recursively refines with a more intensive strategy until success or $z=\textsf{high}$ is reached, at which point UserError is returned.

### Training and optimization

The training of TriSQL follows a coordinated multi-stage strategy that balances the specialization of individual components with end-to-end integration. Each module is first optimized with tailored objectives to learn its specific functionality, after which joint fine-tuning aligns their behaviors to ensure seamless interaction across the framework.

The Question-Guided Schema Selector is trained to maximize the accuracy of table and column relevance prediction. Since relevant elements are typically sparse compared to irrelevant ones, we employ focal loss to focus on hard-to-classify cases and mitigate class imbalance:

12 $$\begin{aligned} \mathscr {L}_{\text {sel}} = \frac{1}{N}\sum _{i=1}^{N} FL(y_i^t, \hat{w}^{(t)}_i) \;+\; \frac{1}{M}\sum _{j=1}^{M} FL(y_j^c, \hat{w}^{(c)}_{ij}), \end{aligned}$$

where $y_i^t$ and $y_j^c$ are ground-truth relevance labels for tables and columns, and $\hat{w}^{(t)}_i$, $\hat{w}^{(c)}_{ij}$ are the predicted attention-based relevance scores. This design ensures that the selector can effectively highlight the minimal schema subset $S_{\text {ctx}}$ required for generation.

The Structure-Aware SQL Generator is trained with a dual-level objective to capture both structural planning and content instantiation. Specifically, we supervise the generation of structural sequences $y_{\text {struct}}$ as well as the final instantiated SQL $y_{\text {sql}}$:

13 $$\begin{aligned} \mathscr {L}_{\text {gen}} = \lambda _{\text {struct}} \mathscr {L}(y_{\text {struct}}, \hat{y}_{\text {struct}}) \;+\; \lambda _{\text {sql}} \mathscr {L}(y_{\text {sql}}, \hat{y}_{\text {sql}}), \end{aligned}$$

where $\mathscr {L}(\cdot )$ denotes a cross-entropy sequence loss. This encourages the generator to first model a coherent clause-level structure and then fill it with schema-grounded content, reducing syntactic errors and improving logical consistency.

The Complexity-Aware SQL Refiner is optimized in two stages. First, supervised fine-tuning on complexity-labeled pairs $(q, y_{\text {sql}}^{\text {init}}, y_{\text {sql}}^{\text {ref}}, z)$ teaches the refiner to perform different refinement behaviors under different complexity levels $z \in \{\textsf{low}, \textsf{medium}, \textsf{high}\}$. Second, reinforcement learning with execution feedback directly optimizes task-level performance. We define the reward as:

14 $$\begin{aligned} R(y_{\text {sql}}^{\text {out}}) = \alpha \cdot \mathbb {I}\big [V(y_{\text {sql}}^{\text {out}}).\text {exec}=1\big ] \;+\; \beta \cdot \text {Sim}(y_{\text {sql}}^{\text {out}}, q), \end{aligned}$$

where the first term rewards executable SQL and the second term $\text {Sim}(\cdot )$ measures semantic alignment with the question *q*. The RL objective minimizes the negative expected reward:

15 $$\begin{aligned} \mathscr {L}_{\text {ref}} = -\mathbb {E}_{y_{\text {sql}}^{\text {ref}} \sim \pi _\theta }\,[\,R(y_{\text {sql}}^{\text {out}})\,], \end{aligned}$$

where $\mathbb {E}_{y_{\text {sql}}^{\text {ref}} \sim \pi _\theta }[\cdot ]$ denotes the expectation over refined SQL candidates sampled from $\pi _\theta (\cdot \mid q, y_{\text {sql}}^{\text {init}}, S_{\text {ctx}}, z)$. Since the SQL output space is large and discrete, this expectation is intractable to compute exactly and is therefore estimated via Monte Carlo sampling by averaging rewards over $N{=}5$ sampled candidates. For each sampled $y_{\text {sql}}^{\text {ref}}$, the fallback mechanism selects the final output $y_{\text {sql}}^{\text {out}}$, which is then used to compute the execution-based reward $R(\cdot )$.

After pre-training each module separately, we jointly fine-tune them so that they work together as one pipeline. We use complexity-aware data augmentation to cover SQLs at low, medium, and high complexity levels. With this multi-stage training, TriSQL not only learns the strengths of each module but also delivers stable end-to-end performance on SQLs of different complexity.

## Experiment

### Experimental setup

Our experiments mainly focus on generating SQL SELECT queries, as our target deployment scenario in the business system is centered on read-only, information-seeking analytics, and the standard public Text-to-SQL benchmarks (including Spider) likewise restrict evaluation to SELECT-style retrieval queries. We conducted experiments on the Spider dataset and three widely used variants: Spider-DK, Spider-Syn, and Spider-Realistic^3,9,23,62^. Spider is a large-scale, cross-domain benchmark and the standard for Text-to-SQL evaluation. It contains 10,181 natural language questions and 5,693 unique SQL queries across 200 databases in 138 domains. Several variants introduce extra challenges: Spider-DK adds external domain knowledge to test cross-domain generalization, Spider-Syn increases syntactic diversity to assess robustness in parsing and generation, and Spider-Realistic provides SQL queries that resemble real-world cases with higher structural and semantic complexity.

We evaluate our model using Exact Match (EM) and Execution Accuracy (EX), which are standard metrics in Text2SQL research. Formally, given a reference SQL $y_{\text {reference}}$ and a predicted SQL $y_{\text {sql}}$, EM is defined as

16 $$\begin{aligned} \textrm{EM} = \frac{1}{N}\sum _{i=1}^{N} \mathbb {I}\big [y_{\text {sql}}^{(i)} = y_{\text {reference}}^{(i)}\big ], \end{aligned}$$

where $\mathbb {I}[\cdot ]$ is the indicator function, returning 1 only if the predicted SQL $y_{\text {sql}}^{(i)}$ matches the reference $y_{\text {reference}}^{(i)}$ exactly at both the structural and content level, i.e., all clauses, operators, schema elements, and literal values are identical; otherwise it returns 0. Execution Accuracy instead compares the execution results of the predicted SQL and the reference SQL on the database $\mathscr {D}$:

17 $$\begin{aligned} \textrm{EX} = \frac{1}{N}\sum _{i=1}^{N} \mathbb {I}\big [\textsf{Exec}(y_{\text {sql}}^{(i)}, \mathscr {D}^{(i)}) = \textsf{Exec}(y_{\text {reference}}^{(i)}, \mathscr {D}^{(i)})\big ]. \end{aligned}$$

We use EX as the primary evaluation metric because it directly shows whether the generated SQL can run correctly on a real database. In contrast, EM may underestimate performance when semantically equivalent but syntactically different SQL queries are produced. In fact, many recent approaches report high EM but considerably lower EX, indicating that the generated SQL often match the reference standard at string-level but still fail to execute correctly. For practical deployment, achieving higher EX is more desirable, since reliable execution accuracy directly determines whether a Text2SQL system can be trusted in real-world applications.

To validate TriSQL beyond SELECT-only settings, we construct PowerSQL, a power-domain benchmark derived from the power dispatching business system of State Grid Jiangsu Electric Power Company. PowerSQL contains 3,427 natural language requests paired with executable SQL statements, collected from real database interactions in power grid management workflows over a 24-month period. The dataset includes 2,741 SELECT queries for analytics and reporting, 274 INSERT statements for logging equipment installations and fault events, 241 UPDATE statements for updating equipment status and maintenance records, 103 DELETE statements for removing obsolete entries, and 68 CREATE TABLE statements for defining new monitoring data structures. The underlying schema comprises 58 tables with complex foreign-key relations, inducing multi-table join paths of up to five tables.

For evaluation on PowerSQL, we introduce extended metrics EM* and EX* to uniformly handle diverse SQL operation types. EM* measures exact string match between the predicted SQL and the reference SQL:

18 $$\begin{aligned} \mathrm {EM*} = \frac{1}{N}\sum _{i=1}^{N} \mathbb {I}\big [y_{\text {sql}}^{(i)} = y_{\text {reference}}^{(i)}\big ], \end{aligned}$$

where $\mathbb {I}[\cdot ]$ equals 1 if the prediction matches the reference exactly in both structure and content, and 0 otherwise. EX* verifies both successful execution and correct database effects:

19 $$\begin{aligned} \mathrm {EX*} = \frac{1}{N}\sum _{i=1}^{N} \mathbb {I}\Big [\textsf{ExecSuccess}\big (y_{\text {sql}}^{(i)}, \mathscr {D}^{(i)}\big )\ \wedge \ \textsf{VerifyEffect}\big (y_{\text {sql}}^{(i)}, \mathscr {D}^{(i)}, \mathscr {C}^{(i)}\big )\Big ], \end{aligned}$$

where $\textsf{ExecSuccess}$ indicates that the SQL executes without errors, and $\textsf{VerifyEffect}$ checks whether the execution produces the intended effect on the database state $\mathscr {D}$ under verification conditions $\mathscr {C}$. Concretely, for SELECT queries, $\textsf{VerifyEffect}$ compares the returned result set with that of the reference query. For INSERT, it verifies that the specified rows are inserted with correct values. For UPDATE, it confirms that the intended rows are modified as expected. For DELETE, it checks that the target rows are removed. For CREATE TABLE, it validates that the table is created with the correct schema definition. This unified protocol ensures that generated SQL is not only executable but also semantically correct across operation types.

All experiments are conducted on a server equipped with an Intel Xeon Gold 6242R CPU with 80 cores and two NVIDIA RTX 4090D GPUs, each with 48 GB of memory.

### Evaluation result

**Table 4:** Exact Match (EM) and Execution Accuracy (EX) comparison on the spider benchmark.

Approach	EM	EX
**Baseline Methods**		
T5-3B	68.1	70.0
RAT-SQL + GAP + NatSQL	68.6	73.3
RASAT + PICARD	70.6	75.7
S^2^SQL + ELECTRA	72.2	-
**Advanced Methods**		
Graphix-3B+PICARD (DB content used)	77.5	74.2
DIN-SQL + CodeX davinci	**57.0**	78.0
RESDSQL-large +NatSQL	76.7	78.2
TriSQL(Ours)	76.4	**82.2**

The results on the Spider test set are shown in Table 4. TriSQL reaches state-of-the-art performance by producing SQL queries that are both accurate and executable. It achieves the highest EX of 82.2% while keeping a competitive EM of 76.4%, showing that it can generate SQL queries that are syntactically correct, semantically faithful, and run successfully on databases.

**Fig. 3:**
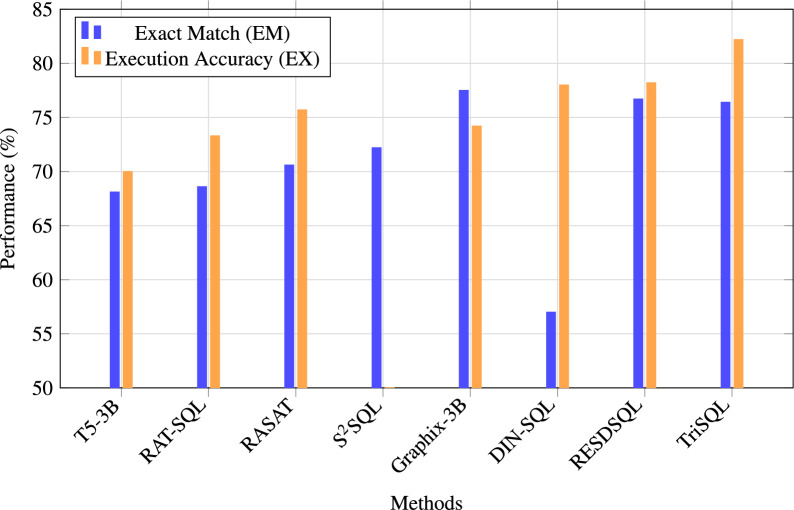
Visualization of EM and EX performance on the Spider benchmark.

To better understand this advantage, Fig. 3 compares TriSQL with representative baselines. Traditional baseline methods such as T5-3B, RAT-SQL + GAP + NatSQL, and RASAT + PICARD achieve EM scores in the range of 68.1 to 70.6, with corresponding EX scores between 70.0 and 75.7^10,11^. Advanced methods exhibit more varied performance characteristics, revealing an inherent trade-off between exact matching and execution robustness. DIN-SQL + CodeX davinci exemplifies this challenge, achieving a high EX score of 78.0% but suffering a substantial decline in EM to 57.0%^21^. This disparity suggests that while the model generates executable queries, they often deviate significantly from the expected ground truth in terms of syntactic structure. Conversely, RESDSQL-large + NatSQL maintains better balance with an EM of 76.7% and EX of 78.2%^45^, yet still fails to achieve optimal performance on both metrics simultaneously. In contrast, TriSQL distinguishes itself by successfully bridging this performance gap. Unlike previous methods that optimize for either syntactic accuracy or execution success, TriSQL achieves superior execution accuracy without compromising exact match performance. The consistent superiority across both metrics demonstrates that TriSQL generates SQL queries that are not only executable but also closely aligned with expected query structures, making it particularly suitable for real-world database applications where both correctness and reliability are essential.

**Fig. 4:**
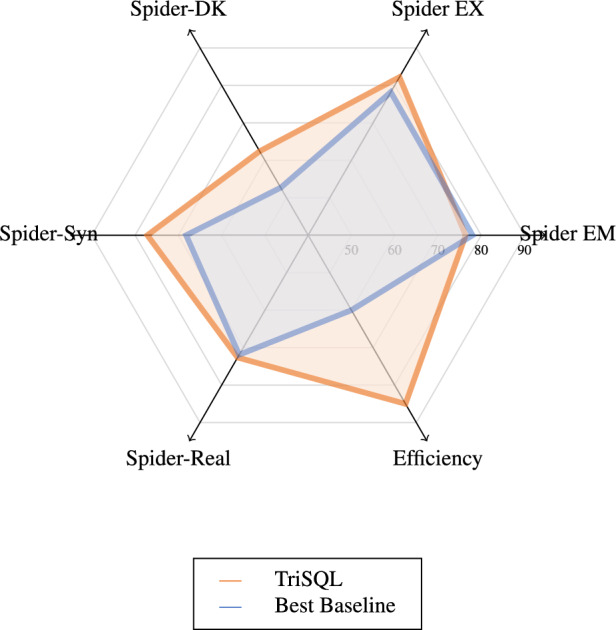
Radar chart comparison of TriSQL with the strongest baseline across six dimensions, including EM, EX, three dataset variants, and computational efficiency.

Beyond the overall results on Spider, we further evaluate TriSQL across multiple dataset variants to assess its robustness under diverse conditions. Figure 4 presents a radar chart comparing TriSQL with the strongest baseline across six dimensions: Spider EM, Spider EX, performance on three challenging variants (Spider-DK, Spider-Syn, and Spider-Realistic), and computational efficiency. This multi-faceted comparison shows that TriSQL consistently surpasses the baseline on most dimensions, with particularly notable gains in execution accuracy and efficiency, while preserving competitive EM performance across all datasets.

**Table 5:** Performance comparison of various approaches on Spider-DK, Spider-Syn, and spider-realistic.

Approach	Spider-DK		Spider-Syn		Spider-Realistic	
	EM	EX	EM	EX	EM	EX
RAT-SQL + BERT	40.9	-	48.2	-	58.1	62.1
RAT-SQL + GRAPPA	38.5	-	49.1	-	59.3	-
T5-3B	-	-	59.4	65.3	63.2	65.0
LGESQL + ELECTRA	48.4	-	64.6	-	69.2	-
TKK-3B	-	-	63.0	68.2	68.5	71.1
T5-3B + PICARD	-	-	-	-	68.7	71.4
RASAT + PICARD	-	-	-	-	69.7	71.9
LGESQL + ELECTRA + SUN	**52.7**	-	66.9	-	**70.9**	-
TriSQL(Ours)	51.02	**62.42**	**72.24**	**77.16**	64.21	**72.63**

**Fig. 5:**
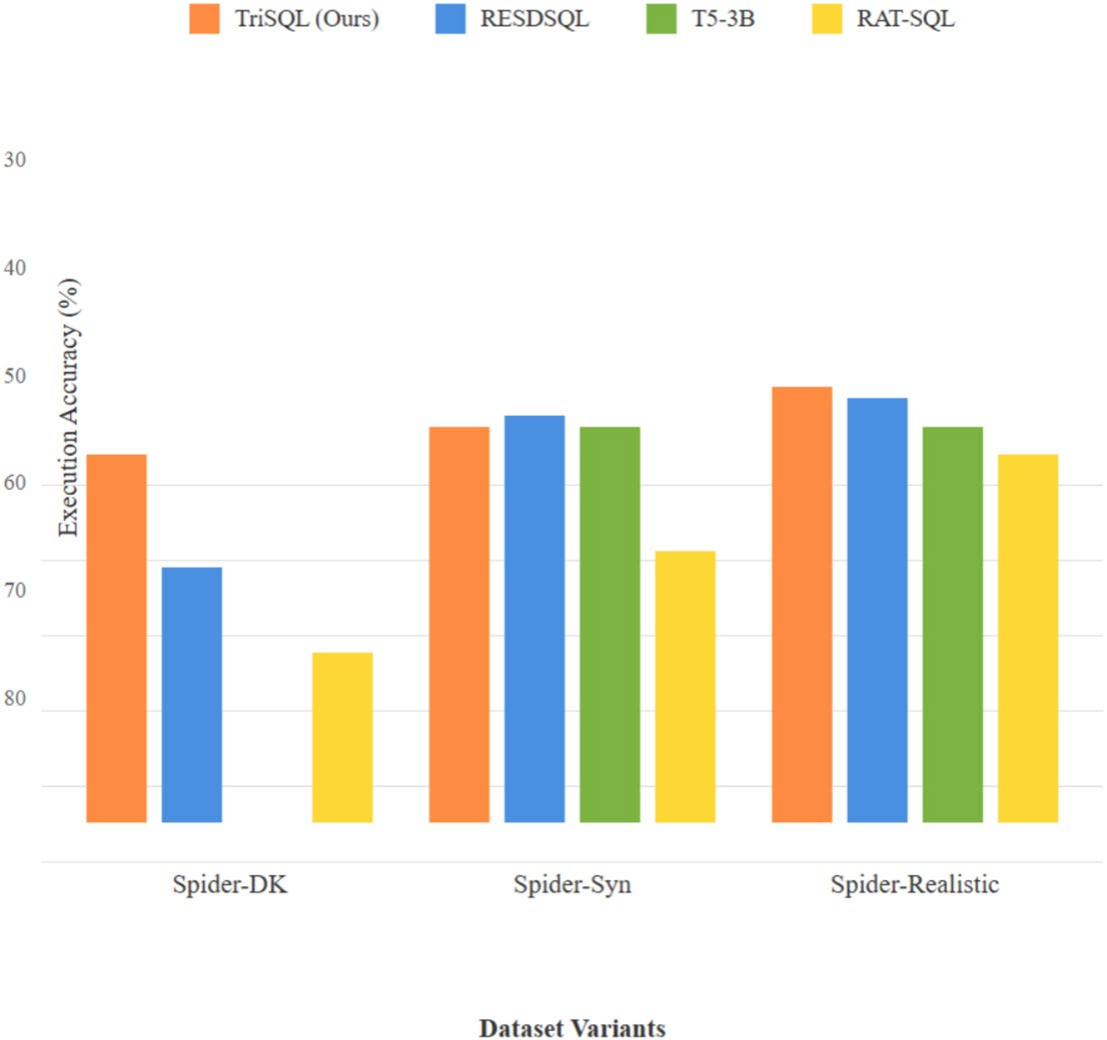
Cross-dataset performance analysis. Direct performance comparison showing TriSQL’s consistent superiority across all dataset variants.

To further examine TriSQL beyond the original Spider benchmark, we evaluate it on three widely used and more challenging variants. Table 5 and Fig. 5 summarize the results, demonstrating TriSQL’s strong generalization ability across diverse settings. On Spider-DK, which tests domain knowledge transfer, TriSQL attains an EM of 51.02 and the highest EX of 62.42, representing an 18.4% improvement over the best baseline (LGESQL + ELECTRA + SUN). On Spider-Syn, which evaluates syntactic robustness, our method achieves the best EM of 72.24 and the best EX of 77.16, surpassing strong baselines such as T5-3B (59.4 EM, 65.3 EX) and TKK-3B (63.0 EM, 68.2 EX) by substantial margins. On Spider-Realistic, TriSQL achieves 64.21 EM and the highest EX of 72.63, showing strong performance on real-world SQL patterns.

We further evaluate robustness under increasing question complexity. Following our robustness definition, we compute a robustness score *R* based on execution accuracy trends across complexity bins. Table 6 summarizes the robustness results for representative baselines and strong recent systems.

**Table 6:** Robustness comparison on Spider.

Approach	Robustness *R*
RAT-SQL + GAP + NatSQL	0.437
RASAT + PICARD	0.443
Graphix-3B + PICARD	0.486
DIN-SQL + CodeX davinci	0.507
RESDSQL-large + NatSQL	0.601
TriSQL (Ours)	0.745

Table 6 compares robustness on Spider using the metric defined in Sec. 3.1. TriSQL achieves the highest robustness score ($R=0.745$), outperforming strong recent systems such as RESDSQL-large + NatSQL ($R=0.601$) and DIN-SQL + CodeX davinci ($R=0.507$). This indicates that TriSQL maintains more stable execution accuracy as question complexity increases, which aligns with its design of combining question-guided schema grounding, structure-first decoding, and complexity-aware refinement to mitigate degradation on hard queries. To further understand how robustness varies across different query characteristics, we next provide a fine-grained analysis along two dimensions: question complexity and query length.

**Fig. 6:**
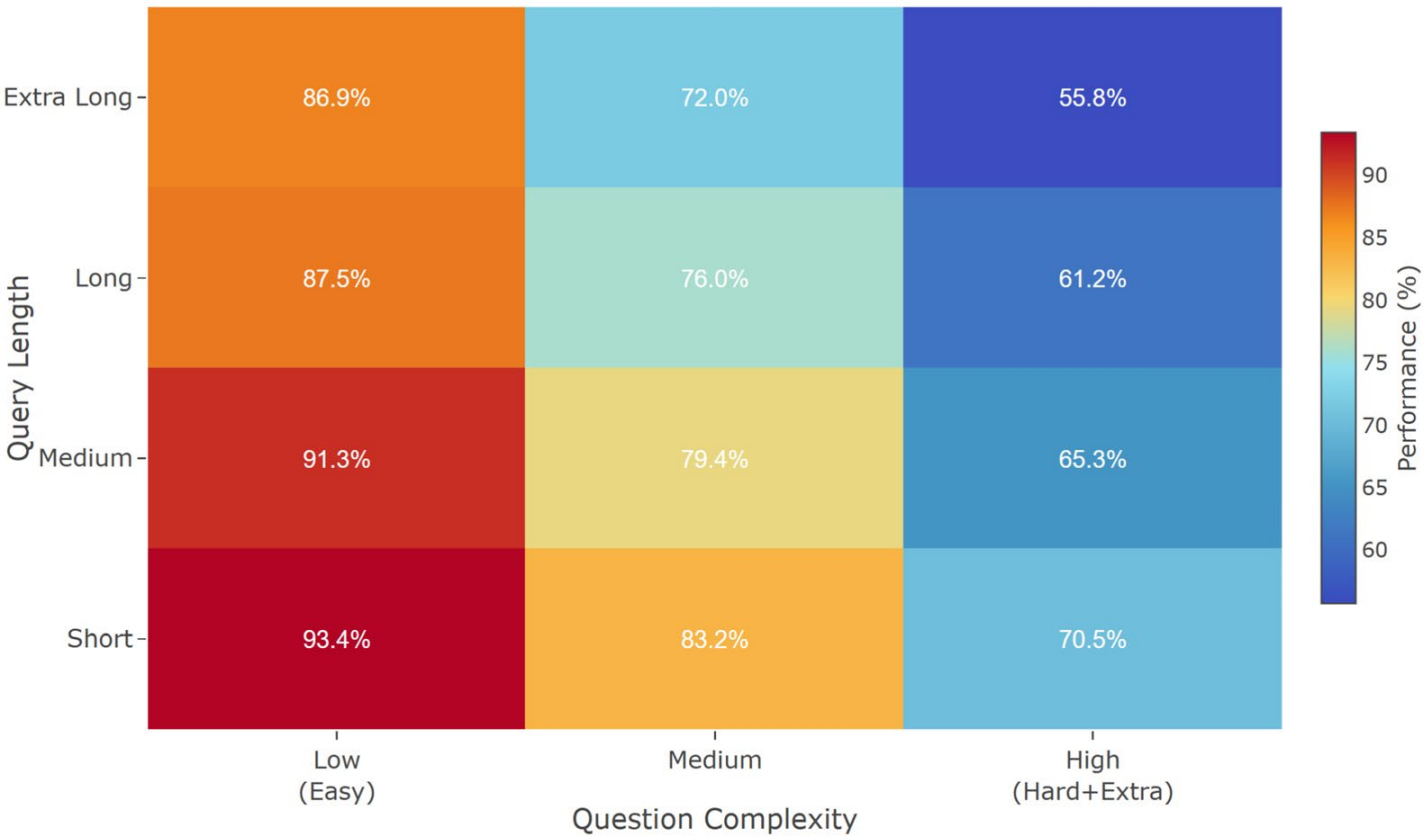
Performance variation of TriSQL across question complexity and SQL length dimensions.

For question complexity, we follow the three-level grouping defined in the Spider benchmark, which includes Low (simple questions whose queries follow basic SELECT, FROM, and WHERE patterns), Medium (queries involving moderate joins and aggregations), and High (Hard and Extra Hard queries that contain complex nesting and set operations, which are particularly challenging because they require generating multiple subqueries with aligned structure and semantics, increasing both syntactic and logical difficulty for NL2SQL models). For query length, we measure the number of major SQL components, including keywords such as SELECT, FROM, WHERE, JOIN, GROUP BY, HAVING, ORDER BY, and LIMIT, as well as subqueries and set operations such as UNION, INTERSECT, and EXCEPT. Based on the natural distribution in the dataset, we further categorize queries into four groups: Short (no more than five components), Medium (six to ten components), Long (eleven to fifteen components), and Extra Long (more than fifteen components). Figure 6 presents the performance distribution of TriSQL across these two dimensions, where warmer colors indicate higher EX. The model achieves its best performance on simpler and shorter queries, while accuracy decreases gradually as either complexity or length increases. Importantly, this degradation remains smooth and stable, without any sharp performance drops even on the most challenging query types. Such consistent behavior demonstrates that TriSQL maintains reliable and predictable performance across varying query characteristics, which is crucial for deployment in real-world applications.

### Efficiency analysis

**Fig. 7:**
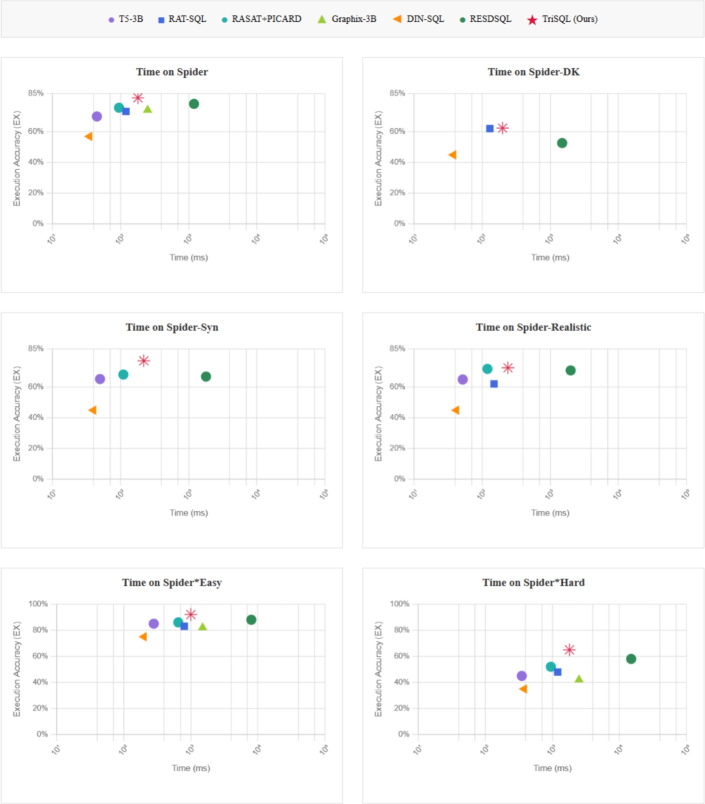
Efficiency comparison with all baseline models. Each subplot illustrates the trade-off between inference time on the x-axis in logarithmic scale and execution accuracy on the y-axis across different datasets. The ideal position lies in the top-left corner, representing high accuracy with low computational cost. The red star indicates TriSQL, showing a balanced trade-off between efficiency and performance.

To evaluate the practical applicability of TriSQL, we conduct a comprehensive efficiency analysis comparing inference time and execution accuracy across multiple datasets with varying complexity levels. Figure 7 presents the trade-off between computational efficiency (measured in milliseconds on a logarithmic scale) and model performance (Execution Accuracy) for seven competing methods across six dataset configurations.

As shown in Fig. 7, the efficiency analysis reveals several key insights. First, TriSQL (represented by red stars) consistently occupies positions in the upper-left region across all datasets, indicating superior execution accuracy with reasonable computational overhead. While DIN-SQL demonstrates the fastest inference times (leftmost positions), it suffers from significantly lower execution accuracy, achieving only 57.0% on the main Spider dataset and dropping to approximately 35-45% on more complex variants. Conversely, RESDSQL achieves competitive accuracy (78.2% on Spider) but requires substantially longer inference times (rightmost positions), often exceeding 10,000 ms on challenging datasets, making it less suitable for real-time applications. On the standard Spider dataset, TriSQL achieves an execution accuracy of 82.2% with an inference time of 180 ms, striking an optimal balance between speed and accuracy. This represents a 4.0 percentage point improvement over RESDSQL while being 6.7$\times$ faster. The efficiency advantage becomes more pronounced on complex datasets. For Spider-DK, which tests domain knowledge transfer, TriSQL achieves 62.42% EX at 200 ms, outperforming all baselines that have available execution data. On Spider-Syn, our method reaches 77.16% EX at 220 ms, demonstrating superior syntactic robustness compared to T5-3B (65.3% at 50 ms) and TKK-3B (68.2% at 110 ms). On the complexity-based subsets of Spider, TriSQL keeps high accuracy on Spider*Easy (about 92%) with moderate latency (1,000 ms). On Spider*Hard, it shows its main advantage: reaching 65% execution accuracy at 1,800 ms, while other methods drop below 60% or take much longer. For example, RESDSQL needs 15,000 ms (8.3$\times$ slower) to get only 58% accuracy on hard SQL queries. These results show that our complexity-aware design uses computational resources more efficiently by adapting to query difficulty. The efficiency of TriSQL comes from three design choices. The Question-Guided Schema Selector reduces the search space early by removing irrelevant schema elements before generation. The Structure-Aware SQL Generator then uses skeleton-based generation to avoid producing SQL queries with invalid syntax. Finally, the Complexity-Aware SQL Refiner applies costly LLM-based refinement only to complex cases, instead of uniformly to all SQL queries. Furthermore, TriSQL shows consistent performance across different datasets, including the syntactically diverse Spider-Syn, the domain-specific Spider-DK, and the real-world Spider-Realistic, which indicates that it can handle different types of SQL complexity. In contrast, methods like Graphix-3B drop sharply on some datasets, and DIN-SQL performs poorly even though it is faster. TriSQL maintains stable performance with a gradual decrease in accuracy, showing strong reliability for practical use across diverse SQL queries.

These efficiency results, together with the execution accuracy improvements shown in the previous section, show that TriSQL is a practical solution for real-world Text-to-SQL applications where both performance and latency matter. On the Spider benchmark, TriSQL achieves state-of-the-art execution accuracy (82.2%) with latency under 1,000ms on standard SQL queries, while still maintaining strong results on complex SQL queries. This balance of accuracy and efficiency makes it well suited for interactive database interfaces and real-time analytics.

### Ablation study

**Fig. 8:**
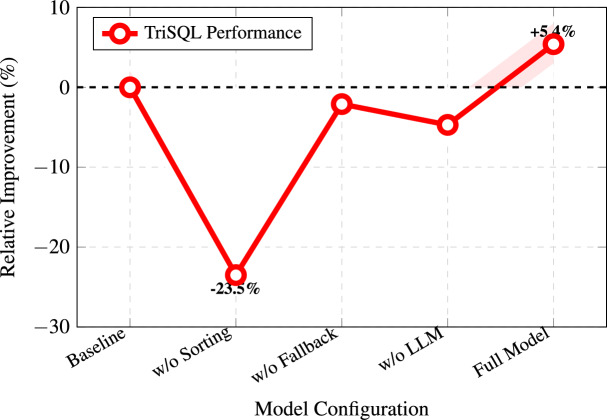
Relative performance improvements from each component in the TriSQL ablation study.

**Table 7:** Percentage improvements of TriSQL over the best baseline across different Spider dataset variants.

Method	Spider-DK	Spider-Syn	Spider-Realistic	Average
TriSQL vs Best Baseline	***+9.72%***	***+11.86%***	***+7.13%***	***+9.57%***

To quantify the contribution of each component in TriSQL, we perform an ablation study by removing one module at a time and measuring the resulting changes in EM and EX. We consider four ablated variants aligned with the key design choices of the framework. The *w/o sorting* variant removes the relevance-based ranking in the Question-Guided Schema Selector (Eq. 3), so schema elements are selected without being prioritized by aggregated relevance scores, weakening question-conditioned schema filtering. The *w/o intelligent fallback* variant disables the execution-aware rollback mechanism in the Complexity-Aware SQL Refiner (Eq. 10), forcing the system to always output the refined SQL even when refinement causes execution failure, which can propagate refinement-induced errors. The *w/o SQL generation* variant removes the skeleton-first, two-phase decoding strategy in the Structure-Aware SQL Generator (Eq. 5–6) and replaces it with standard token-level decoding without explicit structural planning. Finally, the *w/o LLM correction* variant bypasses the complexity-aware refinement procedure (Eq. 7–9) and directly returns the initial draft $y_{\text {sql}}^{\text {init}}$ without refinement-driven semantic correction or execution-guided improvement.

**Table 8:** Performance impact of removing individual components.

Model Variant	EM	EX
- w/o sorting (Eq. 3)	0.5029	0.6266
- w/o intelligent fallback (Eq. 10)	0.524	0.560
- w/o SQL generation (Eq. 5–6)	0.2000	0.1880
- w/o LLM correction (Eq. 7–9)	**0.567**	0.5830
**Full TriSQL Model**	0.764	**0.822**

The results are presented in Table 8 and Fig. 12. Removing the schema sorting mechanism from the Question-Guided Schema Selector causes a substantial performance drop, with EM falling from 76.4% to 50.3% (a 26.1% decrease) and EX declining from 82.2% to 62.7% (a 19.5% decrease). This demonstrates that the relevance-based ranking approach is essential for filtering out irrelevant schema elements. Without proper sorting, the downstream generator receives noisy input containing many unrelated tables and columns, which degrades both syntactic accuracy and execution reliability. Eliminating the intelligent fallback mechanism reduces EM to 52.4% and EX to 56.0%, representing a 26.2% drop in execution accuracy. This confirms that the adaptive rollback strategy plays a critical role in preventing refinement errors from propagating to the final output. When the LLM refinement introduces mistakes, the fallback mechanism can revert to the initial executable SQL, maintaining system reliability.

**Fig. 9:**
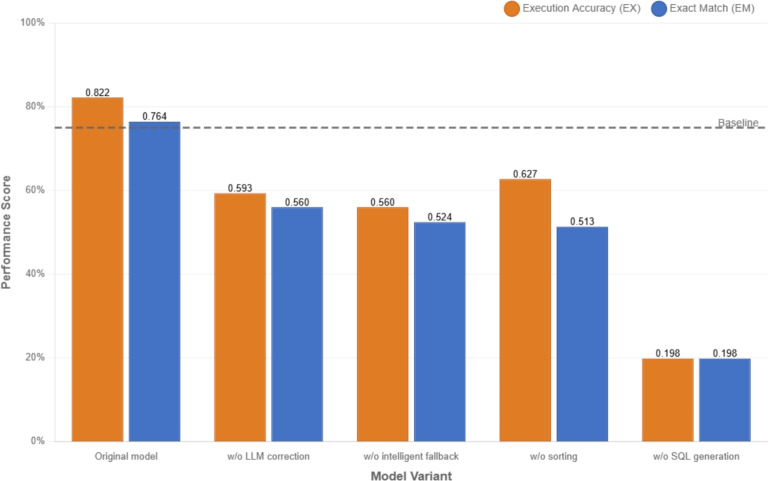
Ablation study results showing the effect of removing individual components from TriSQL. The SQL generation module plays a crucial role, as its removal leads to a substantial drop in performance. The complete model achieves the best overall results through the synergy of all components.

**Fig. 10:**
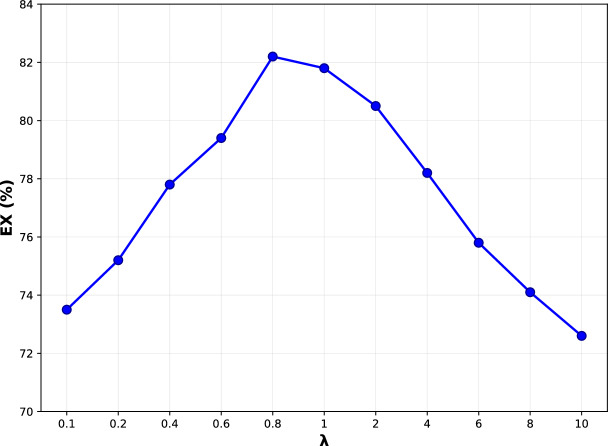
Sensitivity of TriSQL’s EX to the $\lambda$ parameter.

**Fig. 11:**
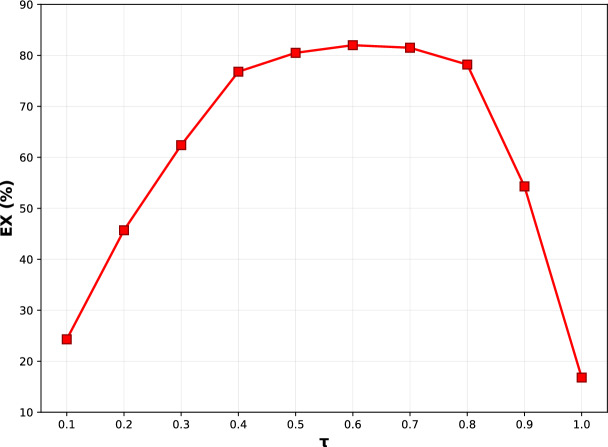
Sensitivity of TriSQL’s EX to the $\tau$ parameter.

To further justify parameter settings in the Question-Guided Schema Selector, we perform a grid search on $\lambda$ and $\tau$, which control table-column attention balance and schema filtering thresholds, respectively. As shown in Figs. 10 and 11, EX peaks at $\lambda =0.8$ (82.2%) and $\tau =0.6$ (82.0%), indicating optimal trade-offs between table-level and column-level relevance and between schema completeness and noise reduction. Performance remains stable within moderate ranges ($\lambda \in [0.4,1.0]$, $\tau \in [0.4,0.8]$), confirming TriSQL’s robustness and practical deployability.

The largest degradation arises when the skeleton-first generation strategy is removed. In this setting, EM drops to 20.0% and EX to 18.8%, i.e., decreases of 56.4 and 63.4 percentage points compared with the full model. This sharp collapse highlights that explicit structural modeling via the two-phase generator (Eq. 5–6) is central to TriSQL. Without separating structure prediction from content instantiation, the model frequently violates SQL syntax and clause composition, yielding malformed queries that fail to execute. Token-level decoding alone is insufficient to preserve the hierarchical and compositional constraints of SQL, especially for complex queries.

Removing the LLM-based complexity-aware refinement yields an instructive contrast: EM slightly increases to 56.7%, while EX declines to 58.3%. This divergence reflects a mismatch between surface-form similarity and functional correctness. Refinement (Eq. 7–9) can modify the SQL string to better align with the database schema and intended semantics, which may reduce exact-match agreement with the reference despite improving executability. This observation further motivates reporting execution accuracy as a primary metric, since it more directly reflects practical utility than exact string matching.

Overall, the full TriSQL pipeline attains the best execution performance (EX 82.2%) while maintaining strong EM (76.4%), indicating that Question-Guided Schema Selection, Structure-Aware SQL Generation, and Complexity-Aware SQL Refinement interact complementarily. The ablation study suggests that each stage targets a distinct failure mode in Text-to-SQL, and their combination is necessary to achieve robust performance across varying levels of query complexity.

We conducted an ablation study to measure the contribution of each component in TriSQL by removing modules one at a time and observing the impact on EM and EX. The results are shown in Table 8. Removing the sorting module causes a large performance drop, with EM falling to 0.5029 and EX to 0.6266, which shows the importance of ranking tables and columns for selecting the right schema and keeping SQL accuracy. Eliminating the fallback mechanism lowers EM to 0.5524 and EX to 0.6056, indicating that this mechanism improves reliability when the initial generation fails. Removing the SQL pre-generation stage leads to the sharpest decline, with EM dropping to 0.2000 and EX to 0.1880, confirming that reference examples are necessary to guide the model in building syntactically and semantically correct SQLs. Without the LLM correction module, EM slightly rises to 0.567 but EX falls to 0.5830, showing that this module is key to execution accuracy even if its edits are small. The complete TriSQL model achieves the highest EX of 0.822 while keeping competitive EM, proving that all components together are needed for both high accuracy and reliable execution.

**Fig. 12:**
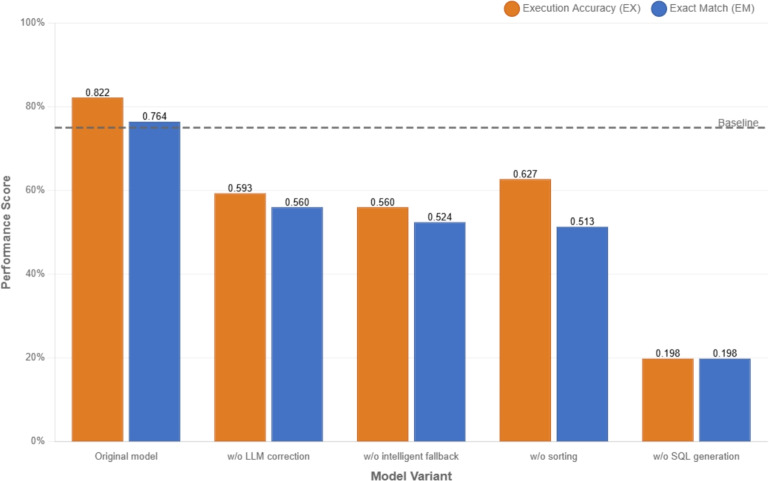
Ablation study results showing the effect of removing individual components from TriSQL. The SQL generation module plays a crucial role, as its removal leads to a substantial drop in performance. The complete model achieves the best overall results through the synergy of all components.

### Evaluation on broader SQL operations

To validate TriSQL beyond SELECT-only settings, we evaluate it on the PowerSQL benchmark. Table 9 reports performance across diverse SQL queries queries operation types.

**Table 9:** Performance on the PowerSQL benchmark across different SQL operation types.

Operation Type	Count	TriSQL		DIN-SQL	
		EM* (%)	EX* (%)	EM* (%)	EX* (%)
SELECT	2,741	82.6	91.4	64.3	82.8
INSERT	274	79.8	93.2	61.2	80.4
UPDATE	241	78.4	90.6	58.7	76.9
DELETE	103	76.9	89.1	55.4	73.2
CREATE TABLE	68	73.5	87.3	52.1	68.5
**Overall**	**3,427**	**81.5**	**90.8**	**63.1**	**81.2**

TriSQL attains an overall EM* of 81.5% and EX* of 90.8% on PowerSQL, substantially outperforming DIN-SQL (63.1% EM*, 81.2% EX*). TriSQL maintains EX* above 87% for all operation types, with EX* exceeding 90% for SELECT, INSERT, and UPDATE. On SELECT queries, TriSQL reaches 91.4% EX*, improving over its Spider performance by 9.2 percentage points. This gain is consistent with PowerSQL being a focused domain, where power-grid terminology maps more consistently to specific schema elements, reducing schema-linking ambiguity relative to Spider’s cross-domain setting. For data manipulation statements, TriSQL remains robust, achieving 93.2% EX* on INSERT, 90.6% on UPDATE, and 89.1% on DELETE. The strong INSERT performance reflects the structured nature of insertion statements and the clear semantic cues in domain-specific requests. For CREATE TABLE, TriSQL achieves 87.3% EX* and 73.5% EM*, outperforming DIN-SQL by 18.8 percentage points in EX*. Schema definition requires inferring column types, constraints, and relations from natural language, making it more challenging than standard data manipulation. In this setting, the Complexity-Aware SQL Refiner is particularly beneficial, as these cases are typically routed to high-complexity refinement with stronger semantic correction.

Overall, the PowerSQL results demonstrate that TriSQL generalizes well to a broad spectrum of SQL operations. The Question-Guided Schema Selector remains effective across statement types, the Structure-Aware SQL Generator accommodates diverse syntactic templates from SELECT to INSERT and CREATE definitions, and the Complexity-Aware SQL Refiner improves reliability via execution-aware validation and fallback to prevent erroneous modifications. These results support TriSQL as a practical framework for comprehensive database workflows in real deployments.

### Adaptation to a graph DSL

Cypher is a graph query DSL that expresses queries via pattern matching over node labels, relationship types, and property keys. To adapt TriSQL to Cypher, we keep the three-stage pipeline and replace the schema representation, structural skeleton, and execution backend accordingly.

**Question-Guided Schema Selector.**

For Cypher, we define the graph schema space as $S_{\text {graph}}=\{L,R,P\}$, where $L=\{l_1,\ldots ,l_n\}$ are node labels, $R=\{r_1,\ldots ,r_m\}$ are relationship types, and $P=\{p_1,\ldots ,p_k\}$ are property keys. We compute label and relationship relevance using question-to-schema attention:

20 $$\begin{aligned} \textbf{w}^{(l)}=\textrm{softmax}\big ([\textrm{Attn}(q,l_1),\ldots ,\textrm{Attn}(q,l_n)]\big ),\quad \textbf{w}^{(r)}=\textrm{softmax}\big ([\textrm{Attn}(q,r_1),\ldots ,\textrm{Attn}(q,r_m)]\big ). \end{aligned}$$

Property-level scores are computed analogously and used to form a filtered subset $S_{\text {graph,ctx}}$ that contains only labels, relationships, and properties relevant to *q*.

**Structure-aware query generator.**

We redefine the structural skeleton $y_{\text {struct}}$ to match Cypher syntax. The skeleton is composed of Cypher clauses such as MATCH, WHERE, RETURN, WITH, and ORDER BY, with typed placeholders:

- $\texttt {<}$NODE_PATTERN$\texttt {>}$, e.g., (n:Label {property: value})
- $$\texttt {<}$$REL_PATTERN$$\texttt {>}$$, e.g.,-[r:TYPE]-$$\texttt {>}$$
- $\texttt {<}$CONDITION$\texttt {>}$ for WHERE predicates
- $\texttt {<}$RETURN_FIELD$\texttt {>}$ for projections or aggregations

Following the same two-phase generation in TriSQL (Eq. 5–6), the model first predicts the clause-level skeleton with placeholders, then fills them using elements from $S_{\text {graph,ctx}}$. This improves syntactic validity and graph-schema grounding for traversal paths.

**Complexity-aware query refiner.**

We retrain the complexity classifier $f_{\text {cls}}$ (Eq. 7) with Cypher-specific indicators such as traversal depth, optional matches, and aggregations. The execution validator $\textsf{Exec}(y_{\text {cypher}},\mathscr {G},\tau )$ (Eq. 8) runs candidate Cypher queries on a Neo4j instance $\mathscr {G}$ under timeout $\tau$. The fallback mechanism (Eq. 10) remains unchanged, reverting when refinement introduces invalid patterns or non-executable traversals.

**Experimental setup and results.**

We construct a de-identified internal benchmark with 428 natural language requests paired with executable Cypher queries in the power dispatching business system. We split data by graph schema to evaluate cross-schema robustness, and report EM and EX using the same protocol as SQL. Table 10 shows that TriSQL-Cypher outperforms prompt-only generation and the variant without structure-aware generation, indicating the benefit of schema selection, execution-aware refinement, and clause-level skeleton planning for Cypher.

**Table 10:** Results on the internal Neo4j Cypher benchmark.

Method	EM (%)	EX (%)
Prompt-only LLM (Cypher)	12.34	11.33
w/o structure-aware generation	30.98	38.65
TriSQL-Cypher (full)	**44.16**	**42.54**

Table 10 summarizes results on our internal Cypher benchmark. Prompt-only generation performs poorly (EM 12.34%, EX 11.33%), reflecting frequent Cypher syntax errors (e.g., unbalanced parentheses and incorrect relationship directions) and graph-schema grounding failures (e.g., using non-existent labels or properties). Removing structure-first generation while keeping the remaining TriSQL stages yields a clear improvement (EM 30.98%, EX 38.65%), indicating that question-guided schema selection and execution-aware refinement already mitigate a large portion of irrelevant element usage and basic execution errors. TriSQL-Cypher achieves the best performance (EM 44.16%, EX 42.54%), showing that clause-level skeleton planning further improves MATCH pattern construction and predicate placement, resulting in more executable queries with correct traversal semantics.

### Adaptation to NoSQL: MongoDB aggregation pipelines

NoSQL query generation poses additional challenges due to flexible schemas and nested document structures. We adapt TriSQL to MongoDB aggregation pipelines as follows.

**Question-guided schema selector.**

MongoDB collections do not enforce a fixed schema, and documents within the same collection may contain heterogeneous fields and nested structures. To enable schema linking, we derive an *operational schema view* $S_{\text {mongo}}$ by sampling documents from collections used in our business system and extracting frequently observed fields, types, and nesting paths. Formally, $S_{\text {mongo}}=\{C_1,\ldots ,C_n\}$, where each collection $C_i$ is associated with a set of field paths $F_i=\{f_i^1,\ldots ,f_i^{m_i}\}$ (e.g., user.profile.name, transactions.amount). We then apply the same cross-attention scoring mechanism (Eq. 1–3) to rank and filter collections and field paths based on question semantics, producing a context schema subset $S_{\text {mongo,ctx}}$. Since field paths may refer to nested objects or arrays, we additionally track basic type and structural attributes (e.g., scalar vs. array, nesting depth) to favor fields that are compatible with the intended aggregation operations.

**Structure-aware pipeline generator.**

MongoDB queries are expressed as aggregation pipelines. A pipeline is a sequence of stages, each performing a transformation such as filtering, grouping, projection, sorting, or joining. We redefine the structural skeleton $$y_{\text {struct}}$$ as a stage-level plan over operators such as $match, $group, $project, $sort, $lookup, and $unwind, with typed placeholders:

- $$\texttt {<}$$COLLECTION$$\texttt {>}$$ for collection names,
- $$\texttt {<}$$FIELD_PATH$$\texttt {>}$$ for field paths (e.g., user.email, orders.items),
- $$\texttt {<}$$FILTER_CONDITION$$\texttt {>}$$ for match criteria,
- $$\texttt {<}$$AGGREGATION_OP$$\texttt {>}$$ for aggregation operators (e.g., $sum, $avg),
- $$\texttt {<}$$JOIN_SPEC$$\texttt {>}$$ for $lookup specifications.

Following TriSQL’s two-phase generation (Eq. 5–6), the model first predicts a coherent stage sequence and inserts placeholders, then instantiates them using elements from $$S_{\text {mongo,ctx}}$$. This structure-first formulation encourages valid stage ordering (e.g., applying $match before $group, and $unwind before aggregating over array elements) and correct field-path referencing (e.g., using the $ prefix in expression contexts).

**Complexity-aware pipeline refiner.**

We retrain the complexity classifier $$f_{\text {cls}}$$ (Eq. 7) using MongoDB-specific indicators, including the number of stages, the presence of $lookup, nested array operations (e.g., $unwind), and complex aggregation expressions. For low-complexity pipelines ($$z=\textsf{low}$$), we apply minor fixes such as adding missing $ prefixes or correcting operator names. For medium complexity ($$z=\textsf{medium}$$), we repair stage ordering and resolve ambiguous field references in $group or $project. For high complexity ($$z=\textsf{high}$$), we invoke an LLM to decompose complex aggregations into intermediate stages and validate execution on a controlled MongoDB instance. The execution validator $$\textsf{Exec}(y_{\text {pipeline}},\mathscr {M},\tau )$$ (Eq. 8) runs the candidate pipeline on database $$\mathscr {M}$$ with timeout $$\tau$$, returning execution status and results. The fallback mechanism (Eq. 10) follows the same principle as SQL and Cypher. If refinement introduces errors (e.g., invalid stage syntax, incorrect field paths, or non-executable joins), the system reverts to the previous candidate or escalates refinement as needed.

**Experimental setup and results.**

We build a de-identified MongoDB benchmark from internal service workflows that use aggregation pipelines for reporting and anomaly inspection in the power-service system (e.g., equipment fault analysis and energy consumption aggregation). The benchmark contains 312 natural language requests paired with executable pipelines. We evaluate with EM and EX, where EX checks successful execution and result equivalence under the same inputs.

**Table 11:** Results on the internal MongoDB benchmark.

Method	EM (%)	EX (%)
Prompt-only LLM (MongoDB)	25.25	24.64
w/o inferred schema view	27.75	26.36
TriSQL-Mongo (full)	**32.58**	**32.38**

As shown in Table 11, prompt-only generation performs poorly (EM 25.25%, EX 24.64%), mainly due to invalid stage ordering (e.g., $group before $match), operator misuse, and field-path grounding errors under semi-structured data (e.g., missing $ prefixes or referencing non-existent nested fields). Adding the operational schema view alone yields only marginal gains (EM 27.75%, EX 26.36%), indicating that schema guidance is insufficient without explicit modeling of pipeline structure. TriSQL-Mongo achieves the best performance (EM 32.58%, EX 32.38%), suggesting that the combination of an operational schema view, stage-level structure-first planning, and execution-guided refinement reduces malformed pipelines and grounding errors. The fallback mechanism further improves robustness by preventing refinement-induced regressions.

Overall, the pilot results on Cypher and MongoDB indicate that TriSQL’s three-stage design remains effective beyond SQL once the schema space, structural skeleton, and execution signal are adapted to the target language. The same core mechanisms therefore provide a general framework for natural language to query-language generation across heterogeneous database paradigms.

## Conclusions

This work presents TriSQL, an LLM-based three-stage framework for Text-to-SQL generation that combines question-guided schema selection, structure-aware SQL generation, and complexity-aware refinement. Our contributions are threefold. First, we identify and analyze key limitations in existing methods, such as poor schema linking, lack of structural control in generation, and low execution reliability on complex SQLs. Second, we design a progressive framework that addresses these issues by selecting schema elements based on question semantics, generating SQLs with a skeleton-first approach to ensure valid structure, and refining outputs with complexity-aware strategies to improve semantic accuracy and execution. Third, we validate TriSQL through extensive experiments on the Spider benchmark and its variants, showing consistent gains in both EM and EX over strong baselines and state-of-the-art methods.

The results show that TriSQL adapts well to SQLs of different complexity, from simple retrievals to nested SQLs with multiple joins and constraints. The ablation study confirms that each module plays an important role, with the SQL pre-generation stage giving a structural backbone and the refinement stage improving execution accuracy. Beyond standard Text-to-SQL benchmarks that focus exclusively on SELECT queries, our evaluation on the PowerSQL benchmark containing 3,427 SQL statements demonstrates that TriSQL effectively generalizes to the full spectrum of database operations including INSERT, UPDATE, DELETE, and CREATE TABLE. The framework achieves 90.8% execution accuracy on this domain-specific benchmark, validating its applicability to practical database management scenarios. TriSQL also generalizes beyond SQL to other query languages, with pilot evaluations on Cypher for Neo4j and MongoDB aggregation pipelines demonstrating that the core three-stage design remains effective once schema representation, structural skeleton, and validation signals are adapted to the target language.

Challenges remain in handling highly ambiguous questions requiring disambiguation mechanisms, adapting to unseen database schemas with minimal examples, and optimizing query performance beyond correctness. In future work, we plan to improve the refinement stage with adaptive prompt design and dynamic strategy selection, expand PowerSQL to include more complex DDL operations and transaction patterns, investigate hybrid approaches combining generation with query optimization techniques, and extend the framework to support conversational multi-turn interactions where users can iteratively refine queries through natural language feedback.

## Data Availability

The code and data used in this study are publicly available at https://github.com/taoyushi/SQL-Project, and the Spider dataset is available at https://yale-lily.github.io/spider.
